# Convergent evolution in the mechanisms of ACBD3 recruitment to picornavirus replication sites

**DOI:** 10.1371/journal.ppat.1007962

**Published:** 2019-08-05

**Authors:** Vladimira Horova, Heyrhyoung Lyoo, Bartosz Różycki, Dominika Chalupska, Miroslav Smola, Jana Humpolickova, Jeroen R. P. M. Strating, Frank J. M. van Kuppeveld, Evzen Boura, Martin Klima

**Affiliations:** 1 Institute of Organic Chemistry and Biochemistry, Czech Academy of Sciences, Prague, Czech Republic; 2 Faculty of Veterinary Medicine, Utrecht University, Utrecht, The Netherlands; 3 Institute of Physics, Polish Academy of Sciences, Warsaw, Poland; University of Maryland, UNITED STATES

## Abstract

Enteroviruses, members of the family of picornaviruses, are the most common viral infectious agents in humans causing a broad spectrum of diseases ranging from mild respiratory illnesses to life-threatening infections. To efficiently replicate within the host cell, enteroviruses hijack several host factors, such as ACBD3. ACBD3 facilitates replication of various enterovirus species, however, structural determinants of ACBD3 recruitment to the viral replication sites are poorly understood. Here, we present a structural characterization of the interaction between ACBD3 and the non-structural 3A proteins of four representative enteroviruses (poliovirus, enterovirus A71, enterovirus D68, and rhinovirus B14). In addition, we describe the details of the 3A-3A interaction causing the assembly of the ACBD3-3A heterotetramers and the interaction between the ACBD3-3A complex and the lipid bilayer. Using structure-guided identification of the point mutations disrupting these interactions, we demonstrate their roles in the intracellular localization of these proteins, recruitment of downstream effectors of ACBD3, and facilitation of enterovirus replication. These structures uncovered a striking convergence in the mechanisms of how enteroviruses and kobuviruses, members of a distinct group of picornaviruses that also rely on ACBD3, recruit ACBD3 and its downstream effectors to the sites of viral replication.

## Author summary

Enteroviruses are the most common viruses infecting humans. They cause a broad spectrum of diseases ranging from common cold to life-threatening diseases, such as poliomyelitis. To date, no effective antiviral therapy for enteroviruses has been approved yet. To ensure efficient replication, enteroviruses hijack several host factors, recruit them to the sites of virus replication, and use their physiological functions for their own purposes. Here, we characterize the complexes composed of the host protein ACBD3 and the ACBD3-binding viral proteins (called 3A) of four representative enteroviruses. Our study reveals the atomic details of these complexes and identifies the amino acid residues important for the interaction. We found out that the 3A proteins of enteroviruses bind to the same regions of ACBD3 as the 3A proteins of kobuviruses, a distinct group of viruses that also rely on ACBD3, but are oriented in the opposite directions. This observation reveals a striking case of convergent evolutionary pathways that have evolved to allow enteroviruses and kobuviruses (which are two distinct groups of the *Picornaviridae* family) to recruit a common host target, ACBD3, and its downstream effectors to the sites of viral replication.

## Introduction

Enteroviruses are small RNA viruses that belong to the *Enterovirus* genus of the *Picornaviridae* family. They are non-enveloped positive-sense single-stranded RNA viruses with icosahedral capsids, currently consisting of 15 species. Seven enterovirus species (Enterovirus A-D and Rhinovirus A-C) contain human pathogens, such as polioviruses, numbered enteroviruses, echoviruses, coxsackieviruses, and rhinoviruses. They cause a variety of diseases ranging from common cold to acute hemorrhagic conjunctivitis, meningitis, myocarditis, encephalitis, or poliomyelitis [1]. The genome of the enteroviruses encodes the capsid proteins and seven non-structural proteins (named 2A-2C and 3A-3D). The latter carry out many essential processes including genome replication, polyprotein processing, host membrane reorganization, and manipulation of intracellular trafficking. To facilitate these functions, several host factors are recruited to the sites of enterovirus replication through direct or indirect interactions with viral proteins. For instance, the enterovirus non-structural 3A proteins directly bind to the Golgi-specific brefeldin A-resistant guanine nucleotide exchange factor-1 (GBF1) [2] and acyl-CoA-binding domain-containing protein-3 (ACBD3, also known as GCP60) [3].

ACBD3 is a Golgi resident protein involved in the maintenance of the Golgi structure [4] and regulation of intracellular trafficking between the endoplasmic reticulum and the Golgi [5]. ACBD3 is a multidomain protein composed of several domains connected by flexible linkers. Its central glutamine rich domain (Q domain) interacts with the lipid kinase phosphatidylinositol 4-kinase beta (PI4KB) and with the Rab GTPase-activating proteins TBC1D22A and TBC1D22B [6]. The interaction of ACBD3 and PI4KB causes membrane recruitment of PI4KB and enhances its enzymatic activity [7]. The *C*-terminal Golgi-dynamics domain (GOLD) of ACBD3 has been reported to interact with the Golgi integral protein giantin/golgin B1, which results in the Golgi localization of ACBD3 [5]. However, in enterovirus-infected cells, the ACBD3 GOLD domain interacts preferentially with viral non-structural 3A proteins, which causes re-localization of ACBD3 to the sites of virus replication [8].

The role of ACBD3 in enterovirus replication is not yet fully understood. It has been proposed that recruitment of ACBD3 to the sites of viral replication can lead to the indirect recruitment of its interactors and downstream effectors such as PI4KB, a well-known host factor essential for generation of PI4P-enriched membranes suitable for enterovirus replication [9, 10]. The 3A-ACBD3-PI4KB route represents one of the major described mechanisms of PI4KB recruitment to the sites of enterovirus replication [3, 11], although some other mechanisms employing the viral proteins 2BC [12] or 3CD [13] might be involved as well. Moreover, the formation of the 3A-ACBD3-PI4KB complex represents the major described mechanism of PI4KB recruitment to the replication sites of kobuviruses, members of a distinct group of picornaviruses [3, 14–16]. Previously, it has been suggested that PI4P directly recruits the viral RNA-dependent RNA polymerase (3D^pol^) [9]. Further studies, however, revealed that the affinity of PI4P to 3D^pol^ is too weak to attract 3D^pol^ to target membranes by itself, suggesting that other factors may be involved [17]. Notably, PI4P gradients between various membranes can be used for the transport of other cellular lipids against their concentration gradient [18, 19]. The PI4P/cholesterol exchange machinery was implicated in replication of several enteroviruses [12, 20], suggesting that PI4P can be used by the viral machinery as a mediator to prepare membranes with a specific lipid composition suitable for viral replication.

ACBD3 is an important host factor of various enterovirus species [21], however, the structural determinants of its recruitment to the viral replication sites are poorly understood. To date, the structural information about any picornavirus 3A proteins is limited to a solution NMR structure of the uncomplexed poliovirus 3A protein [22] (pdb code 1NG7) and our previously published crystal structure of the aichivirus 3A protein in complex with the ACBD3 GOLD domain [23] (pdb code 5LZ3). Unfortunately, the latter cannot be used for homology modeling of the enterovirus 3A proteins, given the unrelated primary sequences of the enterovirus and kobuvirus 3A proteins, which indicates distinct mechanisms of hijacking ACBD3 by these two groups of viral pathogens.

In this study, we present a structural, biochemical, and biological characterization of the complexes composed of human ACBD3 and the 3A proteins of four representative enteroviruses. The crystal structures revealed the details of the ACBD3-3A interaction, the 3A-3A interaction causing the assembly of the ACBD3-3A heterotetramers, the interaction between the ACBD3-3A complex and the lipid bilayer, and the roles of these interactions in facilitation of enterovirus replication. The comparison of the structures of the ACBD3: enterovirus 3A complexes and the previously known structures of the ACBD3: kobuvirus 3A complexes [23] uncovered a striking convergence in the mechanisms of how the two distinct groups of picornaviruses recruit ACBD3 and its downstream effectors to the sites of virus replication.

## Results

### Diverse enterovirus species use a conserved mechanism to interact with the host ACBD3 protein

**Fig 1:**
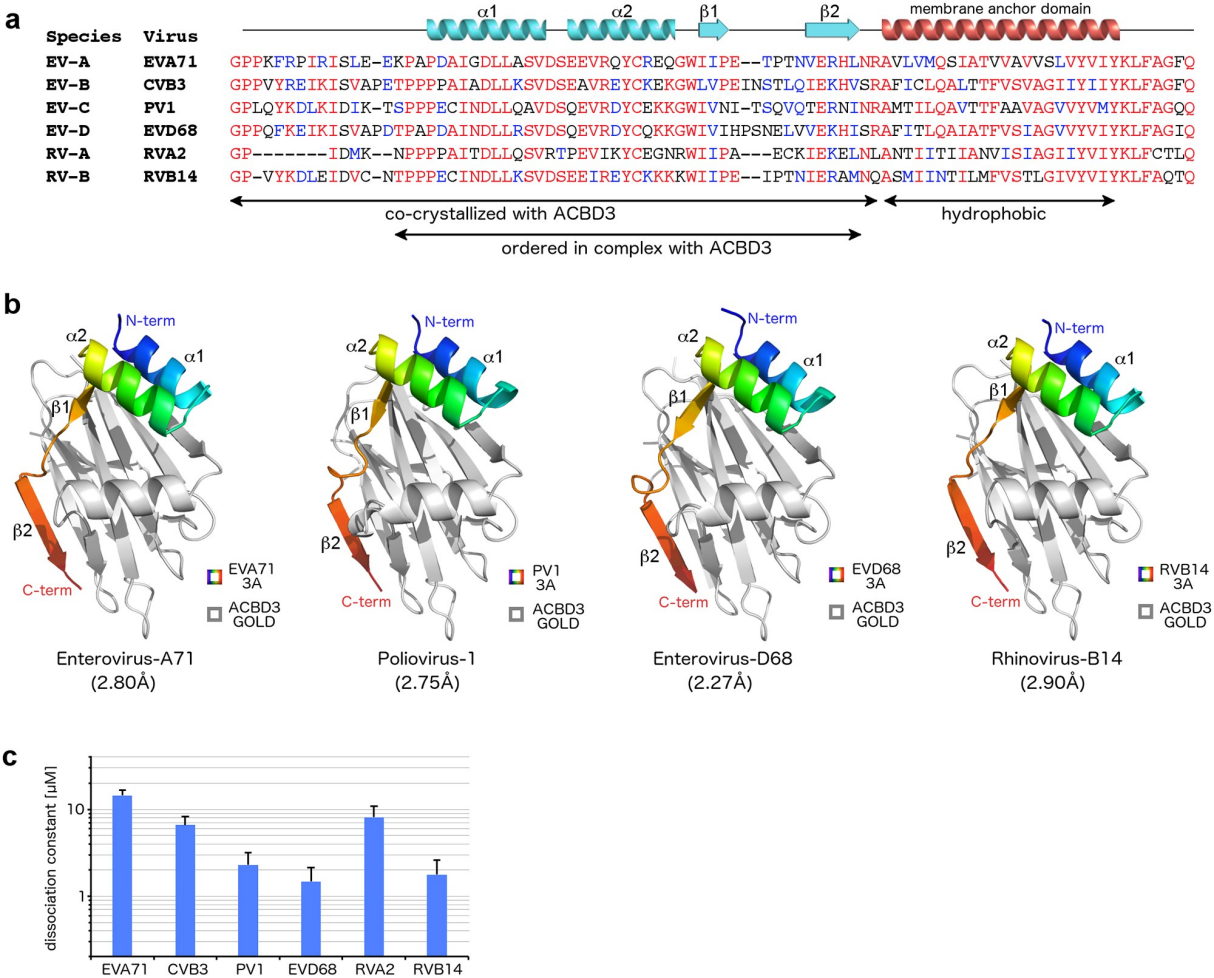
Biochemical and structural characterization of the GOLD: Enterovirus 3A complexes. **a**, Multiple alignment of the 3A proteins of selected enteroviruses used in this study. Sequences were aligned using the ClustalX algorithm and colored using the BoxShade utility. Secondary structures present in the crystal structures of the ACBD3: 3A complexes (colored in light blue) and the hydrophobic alpha helix anchoring the 3A proteins to the membrane (colored in red) are indicated above the sequences. EV, *Enterovirus* species; RV, *Rhinovirus* species; EVA71, enterovirus A71; CVB3, coxsackievirus B3; PV1, poliovirus 1; EVD68, enterovirus D68; RVA2, rhinovirus A2; RVB14, rhinovirus B14. **b**, Overall fold of four different GOLD: enterovirus 3A complexes. The protein backbones are shown in cartoon representation. The ACBD3 GOLD domain is depicted in grey, the viral 3A proteins in rainbow colors ranging from blue (*N* terminus) to red (*C* terminus). **c**, Dissociation constants of the complexes composed of the GB1-fused cytoplasmic domains of the enterovirus 3A proteins and the EGFP-fused ACBD3 GOLD domain as obtained by microscale thermophoresis. Data are presented as mean values ± standard errors of the means (SEMs) from three independent experiments.

For the structural characterization of the enterovirus 3A proteins in complex with the host ACBD3 GOLD domain, we selected 3A proteins of six human-infecting enteroviruses each representing different species as follows: enterovirus A71 (EVA71), coxsackievirus B3 (CVB3), poliovirus 1 (PV1), enterovirus D68 (EVD68), rhinovirus A2 (RVA2), and rhinovirus B14 (RVB14) (Fig 1a).

The recombinant cytoplasmic domains of all the 3A proteins were poorly soluble and tended to aggregate and precipitate at the required concentrations. Therefore, we used 3A proteins *N*-terminally fused to a GB1 solubility tag. For the crystallographic analysis of the complexes composed of the ACBD3 GOLD domain and the viral 3A proteins (hereafter referred to as GOLD: 3A complexes), the GB1-fused cytoplasmic domains of the 3A proteins were directly co-expressed with the ACBD3 GOLD domain in bacteria. The GOLD: 3A complexes were then purified, and the GB1 tag was cleaved off. The GOLD: 3A complexes exhibited better protein solubility than the uncomplexed 3A proteins, sufficient for the subsequent crystallographic analysis.

Of the six GOLD: enterovirus 3A complexes, only GOLD: 3A/EVD68 and GOLD: 3A/RVB14 formed crystals that diffracted to a resolution suitable for subsequent structure determination (i.e. 2.3 Å and 2.9 Å, respectively). Both structures were solved by molecular replacement using a previously published structure of the unliganded ACBD3 GOLD domain (accession number 5LZ1 [23]) as a search model (Fig 1b, Table 1). To improve the crystallization properties of the other four GOLD: 3A complexes, we used two different strategies. The first strategy was based on mutagenesis of selected surface-exposed hydrophobic residues of the 3A proteins to improve the solubility of the respective GOLD: 3A complexes and their capability to be crystallized at higher protein concentrations. This approach led to a successful crystallization of the GOLD: 3A/PV1 complex with an L24A point mutation within the PV1 3A protein. Its structure was then solved at a resolution of 2.8 Å (Fig 1b, Table 1). The second strategy took advantage of the fact that in all three solved GOLD: 3A structures the *C* terminus of the ACBD3 GOLD domain was located in the vicinity of the *N* terminus of the ordered part of the 3A protein. This allowed us to design GOLD-3A fusion proteins with the last residue of ACBD3 (R528^ACBD3^) fused through a short peptide linker (GSGSG) to the first predicted ordered residues of the respective 3A proteins (e.g. K15^3A/EVA71^). This approach led to a successful crystallization of the GOLD-3A/EVA71 fusion protein and its structure solution at a resolution of 2.8 Å (Fig 1b, Table 1). The GOLD: 3A/CVB3 and GOLD: 3A/RVA2 complexes, however, failed to form diffracting crystals even after extensive optimization using both the mutagenesis and fusion-protein strategies.

**Table 1:** Statistics for data collection and processing, structure solution and refinement of the complexes composed of the ACBD3 GOLD domain and 3A proteins of enterovirus A71, poliovirus 1, enterovirus D68, and rhinovirus B14. Numbers in parentheses refer to the highest resolution shell of the respective dataset. R.m.s.d., root-mean-square deviation.

Crystal	GOLD + EVA71 3A	GOLD + PV1 3A	GOLD + EVD68 3A	GOLD + RVB14 3A
Construct	fusion protein	L24A mutant	wild type	wild type
PDB accession code	6HLW	6HLV	6HLN	6HLT
**Data collection and processing**				
Space group	P 21 21 21	C 1 2 1	C 1 2 1	P 1 2 1
Cell dimensions—a, b, c (Å)	46.4, 54.9, 208.7	90.7, 53.8, 62.8	96.9, 55.9, 64.5	54.4, 79.0, 70.6
Cell dimensions—α, β, γ (°)	90.0, 90.0, 90.0	90.0, 107.6, 90.0	90.0, 112.1, 90.0	90.0, 112.4, 90.0
Resolution at I/σ(I) = 2 (Å)	2.80	2.75	2.27	2.90
Resolution range (Å)	48.61–2.73 (2.83–2.73)	43.20–2.50 (2.59–2.50)	47.45–2.10 (2.18–2.10)	42.44–2.82 (2.92–2.82)
No. of unique reflections	14,873 (1,413)	10,009 (983)	18,521 (1,848)	13,308 (1,286)
Completeness (%)	99.62 (98.95)	98.89 (98.89)	98.34 (99.09)	98.38 (95.33)
Multiplicity	5.0 (5.1)	3.4 (3.5)	3.8 (3.8)	3.8 (3.7)
Mean I/σ(I)	12.80 (1.62)	7.43 (1.24)	10.78 (1.21)	9.85 (1.61)
Wilson B factor (Å^2^)	72.87	55.88	47.36	57.90
R-merge / R-meas (%)	7.76 / 8.67	11.71 / 13.98	7.61 / 8.93	10.97 / 12.78
CC1/2	0.998 (0.730)	0.993 (0.542)	0.997 (0.556)	0.995 (0.665)
CC*	1.000 (0.919)	0.998 (0.838)	0.999 (0.845)	0.999 (0.894)
**Structure solution and refinement**				
R-work (%)	23.88 (36.97)	21.50 (30.46)	22.07 (36.93)	21.04 (31.80)
R-free (%)	25.68 (44.64)	24.14 (32.46)	25.08 (37.07)	23.94 (37.05)
R.m.s.d.—bonds (Å) / angles (°)	0.003 / 0.73	0.003 / 0.82	0.007 / 1.06	0.008 / 1.02
Average B factors (Å^2^)	75.8	52.0	47.7	55.2
Clashscore	0.96	0.74	1.08	0.55
Ramachandran favored/outliers (%)	98 / 0	99 / 0	100 / 0	98 / 0

The overall structures of all solved GOLD: 3A complexes are highly similar to each other. This suggests that neither the L24A mutation in the GOLD: 3A/PV1 complex nor the fusion-protein strategy used for the GOLD: 3A/EVA71 complex affected the overall fold of the complexes (Fig 1b). No electron density was observed for the *N* termini of the 3A proteins (approximately the first 15 residues) and we, therefore, assume that this region is intrinsically disordered. This part of the 3A proteins has been previously reported to be involved in the interaction with another host factor GBF1 [2] or it is largely absent (e.g. RVA2) (Fig 1a).

In order to determine the strength of the interaction between the ACBD3 GOLD domain and multiple enterovirus 3A proteins *in vitro*, we used microscale thermophoresis (Fig 1c). The dissociation constants of the GOLD: enterovirus 3A complexes ranged approximately from 1 μM (EVD68 and RVB14) to 15 μM (EVA71).

In summary, our experiments confirmed that the enterovirus 3A proteins interact with the host ACBD3 protein through the GOLD domain of ACBD3 and the cytoplasmic domains of the 3A proteins. These proteins interact directly with dissociation constants within the low micromolar range. Using several approaches, four GOLD: enterovirus 3A complexes were crystallized and their structures were solved. Taken together, these structures document a conserved mechanism how diverse enterovirus species recruit the host ACBD3 protein.

### ACBD3: 3A interaction promotes recruitment of PI4KB and facilitates enterovirus replication

We performed an analysis of the GOLD: 3A interface to identify amino acid residues important for the ACBD3: 3A interaction, co-localization, stimulation of PI4KB recruitment, and facilitation of virus replication in human cells. For this analysis, we chose the GOLD: EVD68 3A complex because we resolved its structure at the highest resolution. Given the high similarity of the various GOLD: enterovirus 3A structures, we assume that the conclusions drawn from the ACBD3: EVD68 3A complex can be applied to the other ACBD3: enterovirus 3A complexes as well.

**Fig 2:**
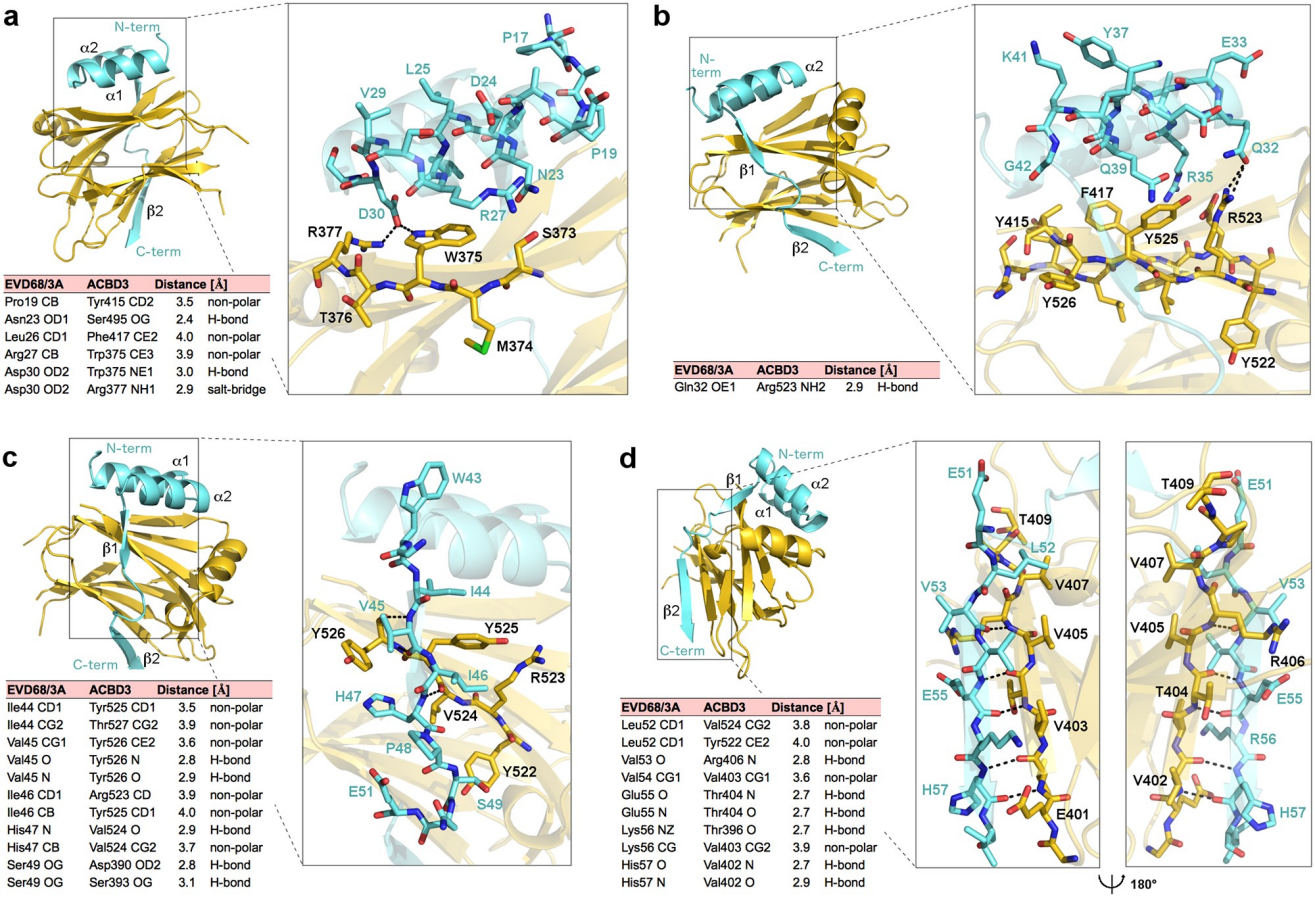
Detailed view of the interface of the GOLD: EVD68 3A complex. **a-d**, Detailed view of the interface between the GOLD domain and the enterovirus-D68 3A protein residues T16-S31 (**a**), S31-G42 (**b**), G42-E51 (**c**), and E51-I58 (**d**). In the overall view, the protein backbones are shown in cartoon representation; the ACBD3 GOLD domain is depicted in gold, the EVD68 3A protein in light blue. In the detailed view, the amino acid residues from the indicated segments are highlighted in stick representation and colored according to elements—oxygen atoms are colored in red, nitrogens in blue, sulfurs in green, carbons according to the protein assignment. Hydrogen bonds are shown as dotted black lines; hydrogen atoms are not visualized. In the lower left of each panel, hydrogen bonds, non-polar interactions, and salt bridges between the GOLD domain and the EVD68 3A protein are listed. The distance cut-off used for hydrogen bonds is 3.3 Å, and for non-polar interactions and salt bridges 4.0 Å. In the case of non-polar interactions, only the closest atom pair for each pair of residues is listed.

In the GOLD: EVD68 3A crystal structure, we could trace the polypeptide chain of the 3A protein from T16^3A^ to I58^3A^. It contains four secondary elements: two alpha helices P19^3A^-V29^3A^ (α1^3A^, Fig 2a) and Q32^3A^-K41^3A^ (α2^3A^, Fig 2b), and two beta strands I44^3A^-I46^3A^ (β1^3A^, Fig 2c) and V53^3A^-I58^3A^ (β2^3A^, Fig 2d). All these segments contribute to the GOLD: 3A interaction mediated through multiple hydrophobic interactions and hydrogen bonds (Fig 2a–2d). The helices α1^3A^ and α2^3A^ bind to a mild cavity of the GOLD domain that is formed by four antiparallel beta strands of ACBD3. The strand β1^3A^ interacts with the strand K518^ACBD3^-R528^ACBD3^ of the ACBD3 GOLD domain, while the strand β2^3A^ binds to the strand V402^ACBD3^-P408^ACBD3^, both in the antiparallel orientation. The conformation of all these secondary elements is highly conserved among various GOLD: enterovirus 3A complexes. The lowest homology of the tertiary structures of these complexes within short linkers between the β1^3A^ and β2^3A^ strands of the 3A proteins corresponds to the lowest homology of the primary sequences of these proteins within this region (Fig 1a and 1b).

**Fig 3:**
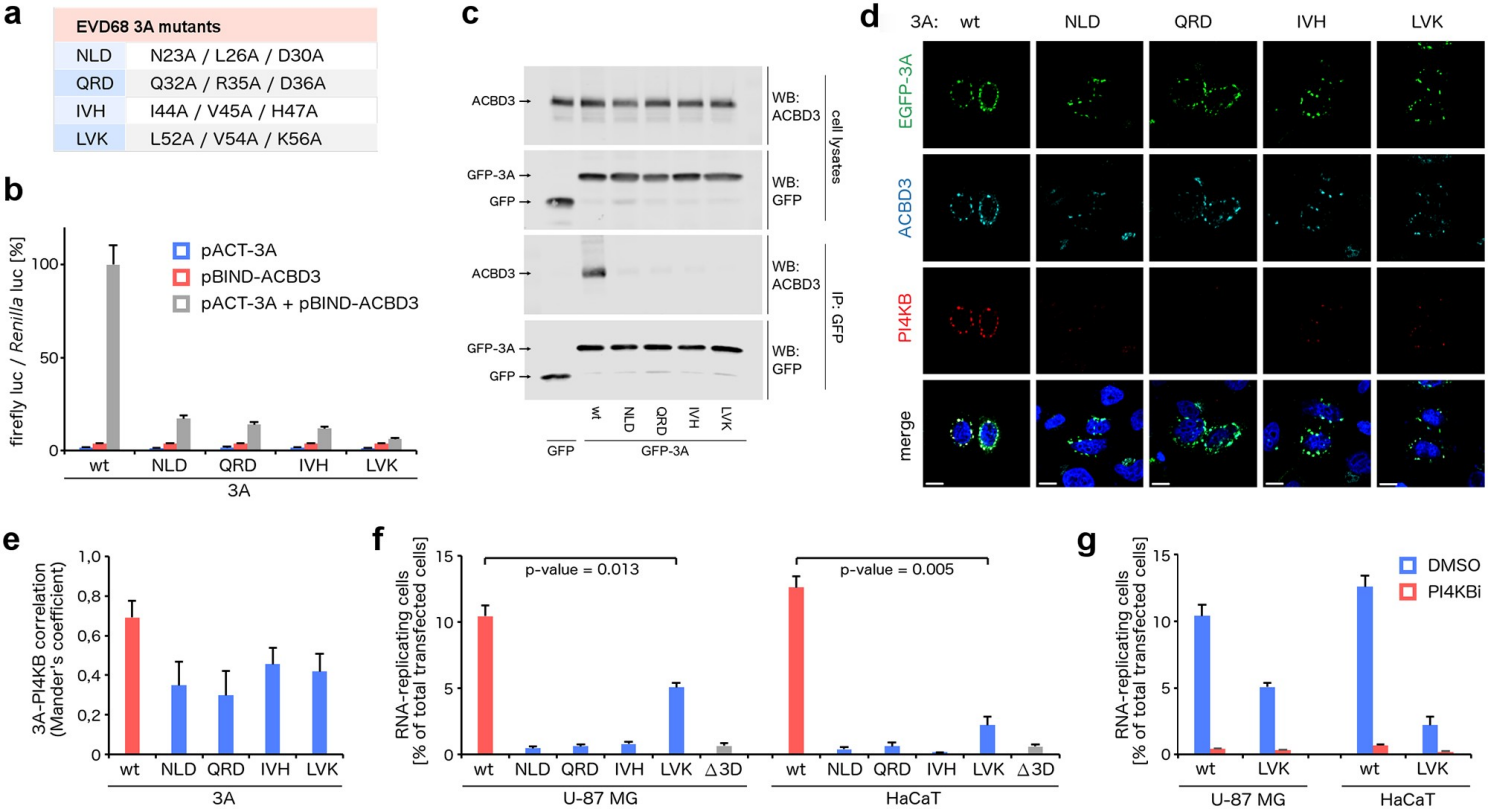
Analysis of the EVD68 3A mutations at the GOLD: EVD68 3A interface. **a**, List of the EVD68 3A mutants designed for further experiments. **b**, Mammalian-two-hybrid assay with the 3A mutants and wild-type ACBD3. HeLa cells were transfected as indicated and the firefly luciferase activity normalized to the *Renilla* luciferase activity was determined using a dual-luciferase reporter assay system. **c**, Co-immunoprecipitation of the 3A mutants and endogenous ACBD3. EGFP-fused wild-type 3A and its mutants were overexpressed in HEK293T cells. The 3A complexes were affinity captured by the GFP-Trap nanobody and resolved by immunoblotting as indicated. **d-e**, Co-localization of the 3A mutants with endogenous ACBD3 and PI4KB. EGFP-fused wild-type 3A and its mutants were overexpressed in HeLa cells. The cells were fixed and immunostained with anti-ACBD3 and anti-PI4KB antibodies. In (**d**), immunofluorescence images of representative cells are shown; scale bars represent 10 μm. In (**e**), the statistical analysis of the 3A-PI4KB co-localization is presented as Mander's correlation coefficients ± standard deviations (SDs) from at least 12 cells from 2 independent experiments. **f-g**, Viral subgenomic replicon assay. U-87 MG and HaCaT cells were transfected with the T7-amplified EVD68 subgenomic replicon wild-type RNA or its mutants as indicated, and the percentage of cells with the reporter mCherry fluorescence above background was determined by flow cytometry. The viral polymerase-lacking mutant (Δ3D) was used as a negative control. In (**f**), cells from the indicated samples were pretreated with a PI4KB-specific inhibitor prior to the transfection of RNA. The data are presented as means ± SEMs from 2 independent experiments.

Calculations [24] of the changes of the interaction energies of various to-alanine mutants of these complexes based on their crystal structures uncovered that multiple amino acid residues of both 3A proteins and ACBD3 are involved in the interaction (S1 Fig). To evaluate the relative importance of various segments of the 3A protein on the complex formation, we designed the following EVD68 3A mutants: NLD (N23A/L26A/D30A), QRD (Q32A/R35A/D36A), IVH (I44A/V45A/H47A), and LVK (L52A/V54A/K56A) (Fig 3a; S1 Fig, panel a). For all the mutants, the ACBD3: 3A interaction was significantly attenuated both in the mammalian-two-hybrid assay (Fig 3b) and in the co-immunoprecipitation assay (Fig 3c), confirming that all four segments of the 3A protein are important for the ACBD3: 3A interaction. Nevertheless, some residual affinity of the 3A mutants to ACBD3 was still observed. All the 3A mutants co-localized with endogenous ACBD3 in the Golgi as did the wild-type 3A protein. The lipid kinase PI4KB, however, was recruited to the Golgi significantly more effectively in the cells expressing wild-type 3A compared to the cells expressing the 3A mutants (Fig 3d and 3e). Under physiological conditions, PI4KB cycles between the cytoplasm and Golgi, where it is recruited by a direct interaction with ACBD3 [7]. In enterovirus-infected cells, the viral 3A protein has been proposed to promote the ACBD3: PI4KB interaction [11]. Thus, considering that no direct interaction between the enterovirus 3A proteins and PI4KB has ever been observed, our data indicate that the stimulation of the ACBD3: PI4KB interaction by the 3A protein and the subsequent increase of the PI4KB recruitment to target membranes in infected cells depends on the ACBD3: 3A interaction. The Golgi-localized PI4P lipid was redistributed in the 3A-expressing cells possibly due to the Golgi disintegration caused by 3A overexpression, nevertheless, no significant change in the PI4P levels was observed in the wild-type 3A-expressing cells compared to the mock-transfected or mutant 3A-expressing cells (S2 Fig). Thus, a cooperation with some other viral proteins can be required to increase the PI4KB activity during viral infection.

To analyze the impact of these 3A mutations on enterovirus replication, we established a reporter subgenomic replicon assay for EVD68. To determine the background reporter expression directly from the transfected RNA, we used a viral polymerase-lacking mutant (Δ3D^pol^). Unexpectedly, no significant replication of the wild-type replicon RNA compared to the Δ3D^pol^ mutant was observed in HeLa cells. However, screening of several human cell lines uncovered the U-87 MG glioblastoma cells and HaCaT keratinocytes in which the wild-type replicon RNA significantly replicated. For all analyzed mutants, the viral RNA replication was attenuated in both cell lines (Fig 3f; S3 Fig). We observed no replication of the NLD, QRD, and IVH mutants, and a significantly reduced replication of the LVK mutant. Notably, this mutant was the weakest ACBD3 interactor in both co-immunoprecipitation and mammalian-two-hybrid assays, indicating additional unknown important effects distinct from the strength of the ACBD3-3A interaction affecting virus replication. We tested whether this mutant gained resistance to the PI4KB inhibition, nevertheless, we found that this mutant was still sensitive to a highly specific PI4KB inhibitor (compound 10 in *Mejdrova et al*. [10]) (Fig 3g).

**Fig 4:**
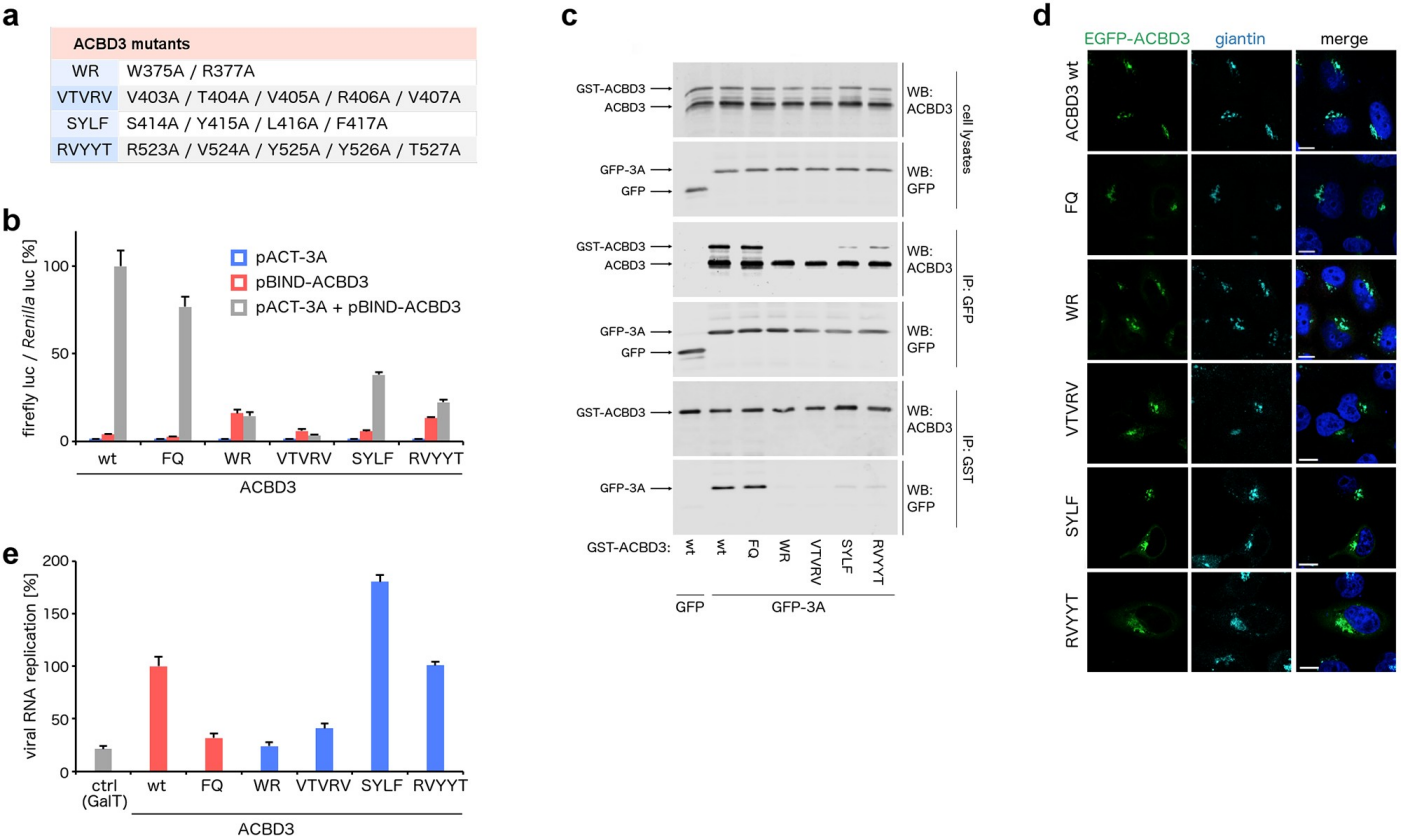
Analysis of the ACBD3 mutations at the GOLD: EVD68 3A interface. **a**, List of the ACBD3 mutants designed for further experiments. **b**, Mammalian-two-hybrid assay with the ACBD3 mutants and wild-type 3A. HeLa cells were transfected as indicated and the firefly luciferase activity normalized to the *Renilla* luciferase activity was determined using a dual-luciferase reporter assay system. **c**, Co-immunoprecipitation of the ACBD3 mutants and wild-type 3A. EGFP-fused wild-type 3A and GST-fused wild-type ACBD3 and its mutants were overexpressed in HEK293T cells. The 3A and ACBD3 complexes were affinity captured by the GFP-Trap nanobody or glutathione sepharose, respectively, and resolved by immunoblotting as indicated. **d**, Localization of the ACBD3 mutants. EGFP-fused wild-type ACBD3 and its mutants were overexpressed in HeLa ACBD3 KO cells. Cells were fixed and immunostained with the anti-giantin antibody. Scale bars represent 10 μm. **e**, Rescue of enterovirus replication by the ACBD3 mutants. HeLa ACBD3 KO cells were transfected with wild-type ACBD3 or its mutants, and enterovirus replication was determined using the *Renilla* luciferase-expressing CVB3 virus by the *Renilla* luciferase assay system. GalT and ACBD3 F258A/Q259A were used as controls.

To address the effect of mutagenesis of selected residues within ACBD3, we designed the following ACBD3 mutants: WR (W375A/R377A), VTVRV (V403A/T404A/V405A/R406A/V407A), SYLF (S414A/Y415A/L416A/F417A), and RVYYT (R523A/V524A/Y525A/Y526A/T527A) (Fig 4a; S1 Fig, panels b-c). In the mammalian-two-hybrid assay (Fig 4b) and in the co-immunoprecipitation assay (Fig 4c), all these ACBD3 mutants displayed a significantly reduced ability to interact with the 3A protein. A weak yet significant effect was observed for the SYLF and RVYYT mutants, while a strong effect resulting in no detectable interaction in both assays was achieved for the WR and VTVRV mutants. Proper intracellular localization of these ACBD3 mutants was verified by their ectopic expression in ACBD3 knock-out cells derived from HeLa cells by CRISPR/Cas9 technology [21]. All these ACBD3 mutants co-localized with giantin, an integral Golgi protein, which has been proposed to directly recruit ACBD3 to the Golgi [5] (Fig 4d).

Finally, we tested the ability of these ACBD3 mutants to rescue enterovirus replication in ACBD3 knock-out cells. The ACBD3 F258A/Q259A mutant, which does not interact with the lipid kinase PI4KB and cannot rescue virus replication [21], was used as a control. The ACBD3 WR and VTVRV mutants failed to rescue virus replication as expected. However, virus replication was still sufficiently restored by the other tested ACBD3 mutants SYLF and RVYYT (Fig 4e). These data document that the remaining affinity of these ACBD3 mutants to the viral 3A protein is still sufficient to fully facilitate enterovirus replication. Surprisingly, the SYLF mutant supports viral replication significantly better than wild-type ACBD3. It is possible that this mutation affects some other ACBD3 properties, such as its ability to interact with some other (known or unknown) proteins involved in enterovirus replication, nevertheless, the exact mechanism of the enhanced enterovirus replication in the ACBD3 SYLF mutant-expressing cells remains unclear. Compared to the ACBD3 WR mutant, the single mutants W375A and R377A still could rescue virus replication (S4 Fig, panels a-b), indicating that both mutations at the ACBD3: 3A interface are required to sufficiently disrupt the ACBD3: 3A interaction to affect virus replication. Several other tested ACBD3 mutants (such as V403A/V405A/ V407A, Y415A/F417A, and R523A/Y525A/Y526A) displayed a reduced affinity to the enterovirus 3A protein and still were able to restore enterovirus replication (S4 Fig, panels c-d). Alternatively, virus replication can be inhibited by single mutations interfering with a proper intracellular localization of ACBD3 (through ACBD3 misfolding and/or loss of the interaction with giantin) as documented by the E419A mutant (S4 Fig, panels e-f).

In conclusion, our data document that the ACBD3: 3A interaction is essential for enterovirus replication. The viral replication, however, can be facilitated by weakly interacting ACBD3 mutants, provided that they are correctly folded and localized in the Golgi in non-infected cells.

### ACBD3: Enterovirus 3A complexes form heterotetramers with a 2:2 stoichiometry

**Fig 5:**
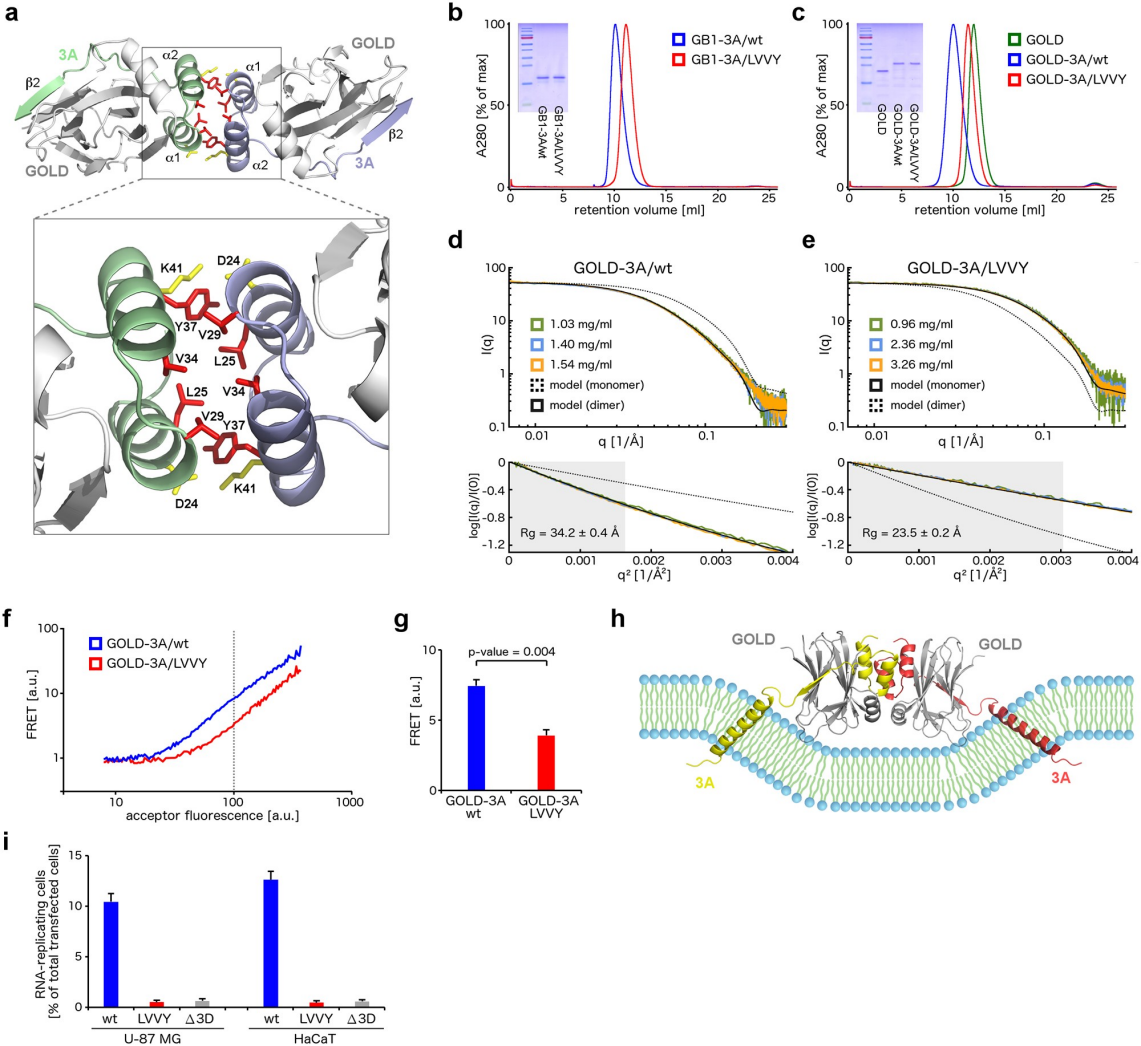
Analysis of the dimerization interface of the GOLD: EVD68 3A complexes. **a**, Overall fold of the heterotetramer composed of two GOLD: 3A complexes (upper panel) and a detailed view of the 3A dimerization interface (lower panel). The ACBD3 GOLD domains are depicted in grey, the EVD68 3A proteins in green and violet. The hydrophobic core of the dimerization interface is highlighted in red and the additional salt-bridge formed by D24 and K41 in yellow. **b**, Elution profiles of the GB1-fused wild-type 3A protein (blue curve) and its LVVY mutant (red curve) in size-exclusion chromatography, monitored by the absorbance at 280 nm. **c**, Elution profiles of the uncomplexed ACBD3 GOLD domain (green curve), GOLD domain fused to the wild-type 3A protein (blue curve) and its LVVY mutant (red curve) in size-exclusion chromatography, monitored by the absorbance at 280 nm. **d-e**, SAXS analysis of the wild-type GOLD-3A fusion protein (**d**) and its LVVY mutant (**e**). In the upper panel, SAXS intensity profiles for three protein concentrations are shown in green, blue, and orange. Structural models (detailed in S7 Fig, panel a) of the dimeric wild-type GOLD-3A fusion protein and its monomeric LVVY mutant yield the scattering curves shown as black solid and dashed lines (**d**) or vice versa (**e**). In the bottom panel, the Guinier plots with the curves colored as in the upper panel are shown. The region of the Guinier approximation valid for globular proteins is shaded in grey. Rg, radius of gyration. **f-g**, FRET analysis of the wild-type GOLD-3A fusion protein and its LVVY mutant. mAmetrine- and mPlum-fusion proteins were transiently co-expressed in HeLa cells and the FRET intensity was determined by flow cytometry. The data are visualized as the FRET signal against a wide range of the acceptor signal from one representative experiment (**f**) or as the mean FRET signal ± SEM at a fixed acceptor signal (marked by a dashed line in (**f**)) from three independent experiments (**g**). **h**, Model of the GOLD: 3A heterotetramer on the lipid bilayer. The ACBD3 GOLD domains are depicted in grey, the EVD68 3A proteins in yellow and red. **i**, Viral subgenomic replicon assay. U-87 MG and HaCaT cells were transfected with the T7-amplified EVD68 subgenomic replicon wild-type RNA or its mutants as indicated, and the percentage of cells with the reporter mCherry fluorescence above background was determined by flow cytometry. The viral polymerase-lacking mutant (Δ3D) was used as a negative control. The data are presented as means ± SEMs from 2 independent experiments.

The enterovirus 3A proteins have been proposed to form homodimers [22, 25]. Analysis of the crystal structures of the GOLD: 3A complexes revealed that the 3A proteins formed either one of the crystal-packing contacts (as in the case of EVD68 and PV1) or contacts with the second 3A molecule when two GOLD: 3A complexes per asymmetric unit were present (as in the case of EVA71 and RVB14). This putative dimerization interface is formed by the two central alpha helices of the 3A proteins, which are bent 180° to form a helical hairpin (Fig 5a). These helices are amphipathic with several hydrophobic residues oriented towards the hydrophobic residues of the other 3A monomer. Surprisingly, the *C* termini of the 3A proteins, which in the cellular environment are anchored to the membranes, are located on the opposite sides of the GOLD: 3A heterotetramers. Therefore, we were interested whether the plasticity and flexibility of the 3A dimerization interface together with the plasticity of the lipid bilayer allows to form the GOLD: 3A heterotetramers at the intracellular membranes.

To identify amino acid residues of the 3A proteins involved in the dimerization of the GOLD: 3A complexes, we calculated [24] the changes of the dimerization energies of various to-alanine mutants of these complexes based on the crystal structures (S5 Fig, panel a). The dimerization interface of the GOLD: 3A/EVD68 complex consists of the hydrophobic core formed by the residues L25, V29, V34, and Y37, and an additional intermolecular salt bridge between the residues D24 and K41 (Fig 5a). To analyze the dimerization of the GOLD: 3A complexes in more detail, we generated a mutant EVD68 3A protein (hereafter referred to as LVVY mutant) with the following four mutations at the putative dimerization interface: L25A, V29A, V34A, and Y37A. As expected, retention volumes of the recombinant wild-type 3A and its LVVY mutant in size exclusion chromatography were significantly shifted corresponding to the dimeric and monomeric state of the wild-type 3A and its LVVY mutant, respectively (Fig 5b).

At the request of a reviewer of our manuscript, we analyzed the dimerization of the L25V, V29Y, L25V/V34L, and V29Y/Y37V mutants (S6 Fig). Both L25V and V29Y mutations attenuated the 3A dimerization. The dimerization of the L25V mutant was restored by the V34L mutation, likely due to the compensation of weakening the L25-L25 interaction by strengthening the V34-V34 and V34-V29 interactions. On the other hand, the potential "rescue" Y37V mutation had a negative impact on the 3A dimerization, likely due to the attenuation of the Y37-L25 interaction and a loss of the hydrogen bond between Y37 and D24 (S6 Fig).

Next, we investigated the stoichiometry of the GOLD: EVD68 3A complexes. To ensure that the 3A protein is fully complexed with the ACBD3 GOLD domain and to avoid the formation of partial complexes with 1:2 stoichiometry, we designed a GOLD-EVD68 3A fusion protein using a similar approach as for the GOLD-EVA71 3A fusion protein used for the crystallographic analysis as described earlier. Taking advantage of the vicinity of the *C* terminus of the ACBD3 GOLD domain and the *N* terminus of the ordered part of the EVD68 3A protein, we connected the last residue of ACBD3 (R528^ACBD3^) through a short peptide linker (GSGSG) to the first ordered residue of the EVD68 3A protein (T16^3A/EVD68^) (S5 Fig, panel b). Both GOLD-3A wild-type and LVVY mutant fusion proteins formed crystals, which diffracted to a resolution suitable for further structure determination (S5 Fig, panel c). The crystal structures of the GOLD: 3A complex consisting of two individual proteins, the GOLD-3A fusion protein, and its LVVY mutant were almost identical with low root-median-square deviations (S5 Fig, panel b), confirming that neither the fusion-protein approach nor the LVVY mutation affected the correct folding of these proteins.

Three lines of evidence document the dimeric state of the wild-type GOLD-3A fusion protein and the monomeric state of its LVVY mutant *in vitro*. First, the retention volumes of these proteins in size exclusion chromatography are significantly shifted (Fig 5c). Secondly, the small-angle X-ray scattering (SAXS) profiles of these proteins correspond to the calculated scattering curves of a dimer of the wild-type GOLD-3A fusion protein (Fig 5d; S7 Fig, panel a) and of a monomer of its LVVY mutant (Fig 5e). Thirdly, crystal contacts corresponding to the 3A dimerization interface are not preserved in the crystal structure of the GOLD-3A LVVY mutant (S7 Fig, panels b-c), indicating that this mutant cannot dimerize through this interface even at very high protein concentrations (of approximately 20 mM) present within the protein crystal.

Next, we investigated the stoichiometry of the GOLD: 3A complexes in cells. For this purpose, we ectopically co-expressed either wild-type GOLD-3A fusion protein or its LVVY mutant *N*-terminally fused to mAmetrine and mPlum fluorescent proteins in HeLa cells and determined the Förster resonance energy transfer (FRET) efficiency by flow cytometry. Owing to the crowding effect, the energy transfer was observed in the case of both proteins. Nevertheless, we observed a significant difference in FRET efficiency between the wild-type GOLD-3A fusion protein and its LVVY mutant (Fig 5f and 5g). These results confirm that the GOLD: 3A complexes are flexible enough to allow the formation of the heterotetramers consisting of two molecules of the viral 3A protein and two molecules of host ACBD3 even in cells at the respective intracellular membranes (Fig 5h).

Finally, we analyzed the impact of the LVVY mutation on enterovirus replication. Using a reporter subgenomic replicon assay for EVD68 established earlier, we found replication of this mutant significantly attenuated in both U-87 MG and HaCaT cells (Fig 5i; S3 Fig). These findings document that the intact dimerization interface of the viral 3A proteins is required for enterovirus replication.

### Proper conformation of the ACBD3: 3A complexes at the membrane is essential for enterovirus replication

**Fig 6:**
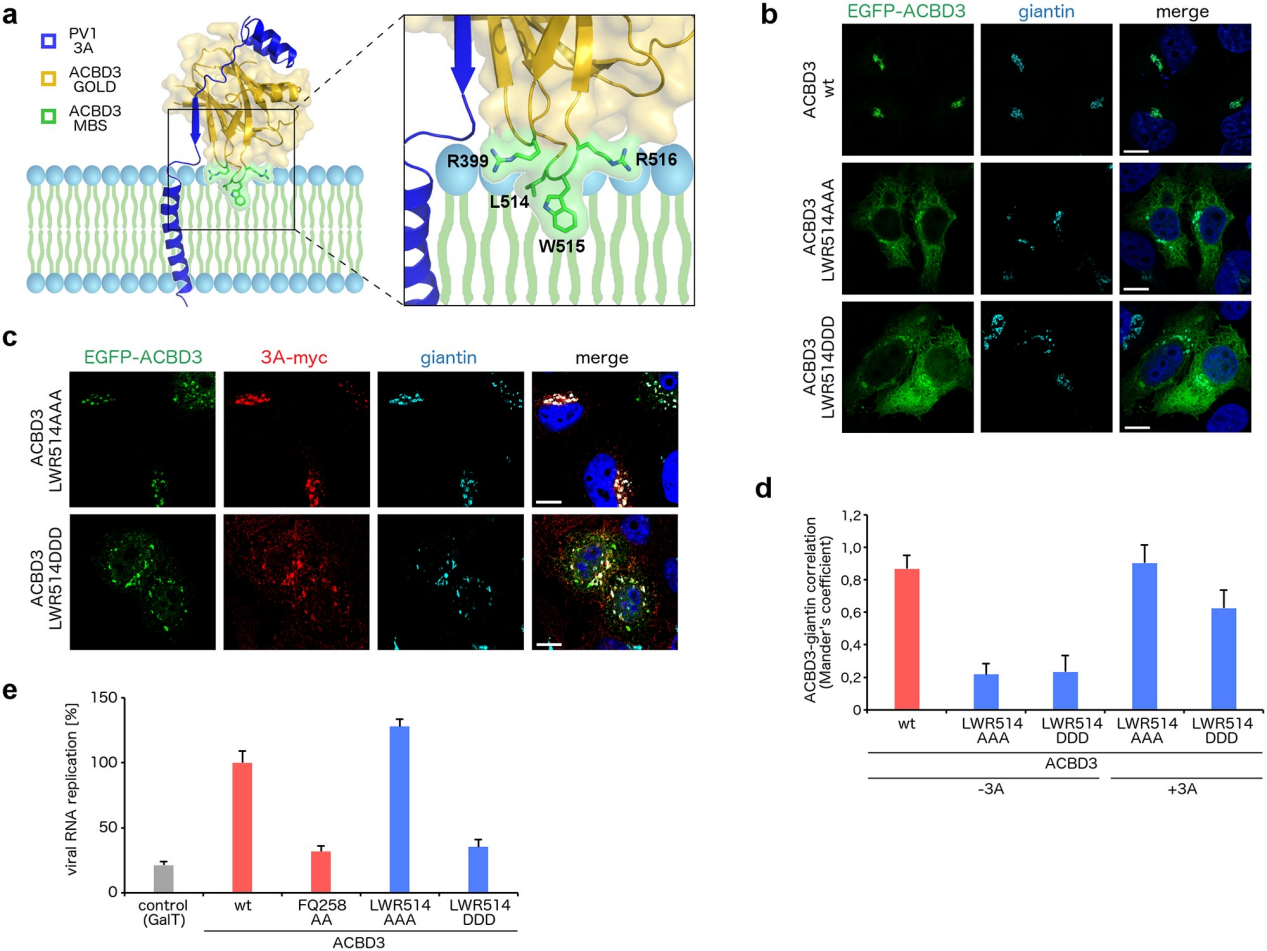
Analysis of the membrane binding site of ACBD3 in complex with the enterovirus 3A protein. **a**, Membrane binding model of the GOLD: poliovirus 3A complex. The ACBD3 GOLD domain is shown in cartoon representation with a semi-transparent surface and colored in gold except for the membrane binding site (MBS) composed of R399, L514, W515, and R516, which is colored in green. The poliovirus 3A protein is depicted in blue. **b**, Localization of the ACBD3 mutants. EGFP-fused wild-type ACBD3 or its mutants were overexpressed in HeLa ACBD3 KO cells. Cells were fixed and immunostained with the anti-giantin antibody (marker of Golgi). Scale bars represent 10 μm. **c**, Localization of the ACBD3 mutants in 3A-expressing cells. EGFP-fused ACBD3 mutants were co-expressed with myc-tagged CVB3 3A in HeLa ACBD3 KO cells. Cells were fixed and immunostained with the anti-myc and anti-giantin (marker of Golgi) antibodies. Scale bars represent 10 μm. **d**, Statistical analysis of the ACBD3-giantin co-localization from (**b**) and (**c**) is presented as Mander's correlation coefficients ± SDs from at least 12 cells from 2 independent experiments. **e**, Rescue of enterovirus replication by the ACBD3 mutants. HeLa ACBD3 KO cells were transfected with wild-type ACBD3 or its mutants, and enterovirus replication was determined using the *Renilla* luciferase-expressing CVB3 virus by the *Renilla* luciferase assay system. GalT and ACBD3 FQ258AA were used as controls.

In a previous study [23], we identified a novel ACBD3 membrane binding site (MBS) consisting of the residues R399, L514, W515, and R516. The hydrophobic residues L514 and W515 can be inserted directly into the lipid bilayer, while the positively charged residues R399 and R516 can interact with the negatively charged phospholipid head groups (Fig 6a). A vicinity of ACBD3 MBS and the expected position of the transmembrane domain of the enterovirus 3A protein within the ACBD3: 3A complexes suggests that ACBD3 MBS may be involved in the stabilization of the ACBD3: 3A complexes at the membrane as well.

To experimentally evaluate this hypothesis, we designed the following ACBD3 mutants with several point mutations within MBS: LWR514AAA and, to increase repulsion between ACBD3 MBS and the lipid bilayer, LWR514DDD. Then, we ectopically expressed wild type ACBD3 or its MBS mutants *N*-terminally fused to EGFP in HeLa ACBD3 knock-out cells. We found that wild-type ACBD3 co-localized with the Golgi marker giantin, while both ACBD3 MBS mutants LWR514AAA and LWR514DDD were mostly released to the cytoplasm, although minor yet significant fractions of their pools were still preserved at the Golgi (Fig 6b). Remarkably, when the ACBD3 MBS mutants were co-expressed with the enterovirus 3A protein, they were completely (LWR514AAA mutant) or partially (LWR514DDD mutant) re-localized back to the Golgi (Fig 6c and 6d). Thus, an intact MBS is required for ACBD3 recruitment to the Golgi by the action of giantin or other cellular factors, however, it is dispensable for ACBD3 stabilization at target membranes through its interaction with enterovirus 3A proteins.

Finally, we tested the capacity of wild-type ACBD3 and its MBS mutants to rescue virus replication in ACBD3 knock-out cells. Both ACBD3 wild type and the LWR514AAA mutant, but not the LWR514DDD and FQ258AA (used as a control [21]) mutants, effectively restored virus replication (Fig 6e). Thus, it seems that not ACBD3 MBS itself but rather the orientation of the ACBD3: 3A complex with respect to the membrane plays a role in facilitation of enterovirus replication. To-alanine mutations of ACBD3 MBS still allow the ACBD3: 3A complex at the membrane to adopt a conformation suitable for viral replication. On the contrary, to-aspartate mutations of ACBD3 MBS, which repel the negatively charged phospholipids of the lipid bilayer, result in an orientation of the ACBD3: 3A complex with respect to the membrane that does not support enterovirus replication.

In summary, ACBD3 MBS is not required for ACBD3 recruitment to target membranes by the action of the enterovirus 3A proteins, however, the proper conformation of the ACBD3: 3A complexes at the membrane mediated by ACBD3 MBS is essential for enterovirus replication.

## Discussion

Considering the commonness of enterovirus-mediated infections within human population, it is surprising that no antiviral therapy for enteroviruses has been approved yet. Targeting essential host factors instead of rapidly mutating viral enzymes represents a promising strategy. Several host factors essential for enterovirus replication are recruited to the sites of viral replication by a direct protein-protein interaction between the host factor and a viral protein. A detailed knowledge of the structures of such complexes can open up prospects for a structure-guided development of small chemical compounds targeting these interactions, yielding a novel class of antivirals to combat infections caused by these pathogens.

In this study, we present a series of crystal structures of complexes composed of the non-structural 3A proteins of four enterovirus species and the 3A-binding GOLD domain of the host factor ACBD3. Previously, the genetic inhibition of ACBD3 mediated by siRNA has yielded conflicting results on the importance of ACBD3 for virus replication [26, 27]. This conflict in the literature has been recently addressed using CRISPR/Cas9-generated ACBD3 knock-out cells, in which enterovirus replication was severely impeded [8, 21]. This confirmed that ACBD3 is, indeed, an essential host factor for enterovirus replication. However, it seems that a very low concentration of ACBD3 within the cells is still fully capable of facilitating enterovirus replication. This hypothesis is in agreement with our observations that enterovirus replication in ACBD3 knock-out cells can be restored by several ACBD3 mutants with a very low affinity to the viral 3A proteins even at the detection limit of conventional methods assessing the protein-protein interactions, such as protein co-immunoprecipitation.

Among picornaviruses, the interaction between the viral 3A protein and host ACBD3 is not unique for enteroviruses. ACBD3 has been proposed to interact also with the 3A proteins of kobuvirus (e.g. aichivirus), hepatovirus, salivirus (klassevirus), and parechovirus, but not with those of cardiovirus (e.g. Saffold virus) or aphthovirus (foot-and-mouth disease virus, FMDV) [6]. To our best knowledge, all picornaviruses sensitive to PI4KB specific inhibitors (such as enteroviruses and kobuviruses) are able to hijack ACBD3, arguing for ACBD3 as a main mediator of PI4KB recruitment by these viruses. Notably, hepatovirus replicates independently of PI4KB [28], indicating either functionally irrelevant interaction with ACBD3 or another, PI4KB-independent, role of ACBD3 in hepatovirus replication. Picornaviruses that cannot hijack ACBD3 through their 3A proteins are either PI4P-independent (such as FMDV [29]) or their replication depends on another PI4P-producing lipid kinase PI4KA (e.g. cardioviruses [30]).

During the past decades, multiple enterovirus mutants resistant to the inhibitors of PI4KB and the oxysterol binding protein (OSBP), which acts downstream of PI4KB, were isolated and characterized. Most of the resistance-conferring mutations were localized to the 3A-encoding regions of these viruses, e.g. PV1 N45Y, R54W, N57D, A70T, and A71S, CVB3 V45A, I54F, and H57Y, and RVB14 E30D/V/Q, I42V, and M54I (S8 Fig) [31–34]. Although many of the PI4KB/OSBP-inhibition resistance-conferring mutations are localized within the ACBD3-interacting regions of the 3A proteins, they seem unlikely to act through modulation of the ACBD3-3A interaction. On the other hand, it is possible that the mutations clustered within the β2 strands of the 3A proteins, such as CVB3 H57Y or PV1 R54W, can act through modulation of the interaction of the ACBD3-3A complex (or the uncomplexed 3A protein) with the membrane. The loss of the positive charge of the mutated residues can possibly compensate for the loss of the negative charge of the PI4P head groups upon PI4KB inhibition. Nevertheless, at least the mechanism of action of the mutations located distally with respect to the membrane, mostly clustered within the β1 strands of the 3A proteins, remains unclear.

**Fig 7:**
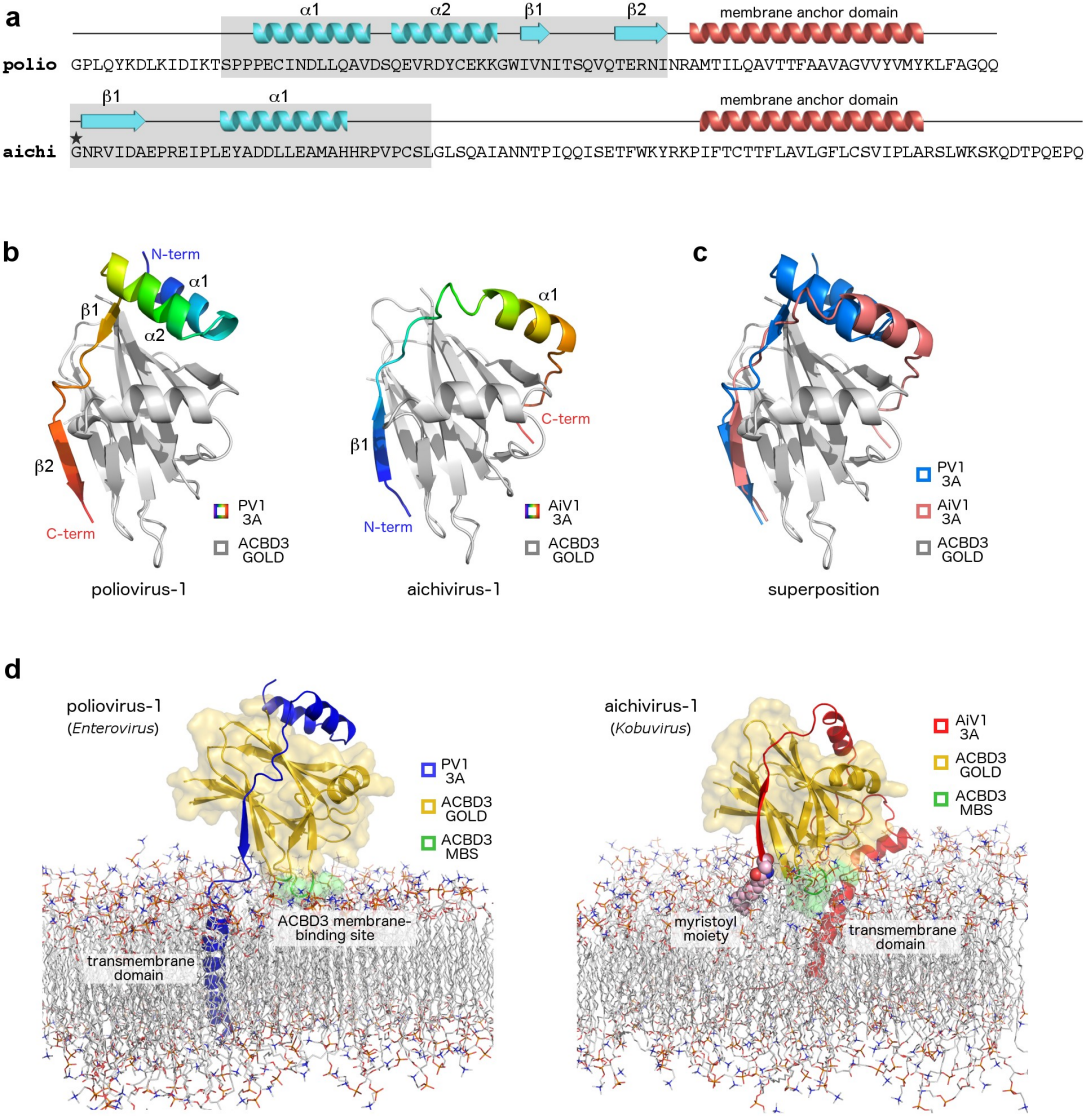
Convergence in the mechanisms of ACBD3 recruitment by enteroviruses and kobuviruses. **a**, Distinct ACBD3-binding regions of enterovirus and kobuvirus 3A proteins. Sequences of the poliovirus-1 (member of enteroviruses) and aichivirus-1 (member of kobuviruses) 3A proteins are shown. Secondary structures present in the crystal structures of the ACBD3: 3A complexes (colored in light blue) and the hydrophobic alpha helices anchoring the 3A proteins to the membrane (colored in red) are indicated above the sequences. ACBD3-binding regions are shaded in grey. Myristoylated Gly1 of the aichivirus-1 3A protein is marked with an asterisk. **b**, Crystal structures of the ACBD3 GOLD domain in complex with the poliovirus (left) and aichivirus (right) 3A proteins. The protein backbones are shown in cartoon representation. The ACBD3 GOLD domain is depicted in grey, the viral 3A proteins in rainbow colors from blue (*N* terminus) to red (*C* terminus). **c**, Superposition of the crystal structures from (**b**). The ACBD3 GOLD domain is depicted in grey, the poliovirus 3A protein in blue, the aichivirus 3A protein in red. **d**, Molecular dynamics simulation-based models of the ACBD3 GOLD domain in complex with the poliovirus (left) and aichivirus (right) 3A proteins on the lipid bilayer. The ACBD3 GOLD domain is shown in cartoon representation with a semi-transparent surface and colored in gold except for the membrane binding site, which is colored in green. The poliovirus 3A protein is depicted in blue, the aichivirus 3A protein in red.

Apart from the crystal structures of the enterovirus 3A: GOLD complexes, to date only the structures of the kobuvirus 3A: GOLD complexes are known [23]. The enterovirus (e.g. poliovirus) and kobuvirus (e.g. aichivirus) 3A proteins share a common overall architecture, i.e. a similar size of approximately 10 kDa, large *N*-terminal soluble cytoplasmic domains followed by hydrophobic membrane-anchoring regions and small *C*-terminal domains (Fig 7a). Despite this common architecture, primary and predicted secondary structures of the enterovirus and kobuvirus 3A proteins are unrelated and cannot be aligned. Furthermore, positions of the ACBD3 binding regions of the enterovirus and kobuvirus 3A proteins are distinct. The ACBD3 binding region of the enterovirus 3A proteins forms the *C*-terminal segments of the cytoplasmic domain (and is preceded by the *N*-terminal GBF1 binding region), while the ACBD3 binding region of the kobuvirus 3A proteins is located at the *N* terminus (and a GBF1 binding region is completely missing). Superposition of the crystal structures of the enterovirus and kobuvirus 3A: GOLD complexes reveals that the enterovirus and kobuvirus 3A proteins bind to the same regions of the ACBD3 GOLD domain, nevertheless, the polypeptide chains of the enterovirus and kobuvirus 3A proteins have opposite polarities (Fig 7b and 7c). For instance, the poliovirus 3A strand β2^3A/PV1^ binds in the antiparallel orientation to the strand V402^ACBD3^-P408^ACBD3^ of ACBD3, while the aichivirus 3A strand β1^3A/AiV1^ binds at the same position to the same strand of ACBD3, but in the parallel orientation (Fig 7b and 7c). The reverse orientation of the enterovirus 3A proteins compared to the kobuvirus 3A proteins may be caused by the specific need of the enterovirus 3A proteins to bind GBF1.

A notable difference between the enterovirus and kobuvirus 3A proteins is represented in the way they are anchored to the membrane. In addition to the *C*-terminal hydrophobic membrane binding regions, kobuvirus 3A proteins are membrane-anchored by the myristoylated *N*-terminal glycines, which is very unusual among picornaviruses [3]. Among enteroviruses, the *N*-terminal myristoylation is not present. To gain more insight into the membrane binding mode of the enterovirus 3A: GOLD protein complex, we performed all-atom molecular dynamics simulation of this complex at the membrane and compared it with our previously published [23] simulation of the kobuvirus 3A: GOLD protein complex at the surface of the lipid bilayer (Fig 7d). These simulations uncovered a similar conformation of the ACBD3 GOLD domain recruited to the membrane by the poliovirus or aichivirus 3A protein, including the insertion of the ACBD3 membrane binding site residues into the lipid bilayer. The position of the *N*-terminal myristoylation of the aichivirus 3A protein functionally substitutes the position of the *C*-terminal transmembrane domain of the poliovirus 3A protein, whereas the *C*-terminal transmembrane domain of the aichivirus 3A protein has no equivalent in the case of the poliovirus 3A protein.

In summary, our findings reveal structural details of how two groups of viral pathogens, enteroviruses and kobuviruses, developed a similar mechanism of hijacking the same host factor (ACBD3) and its downstream effectors (such as PI4KB). These viruses use their 3A proteins with a common architecture yet totally unrelated primary sequences to bind to the same regions of the host ACBD3 protein in the opposite orientations, representing a striking case of convergence in picornavirus evolution. Our results are in agreement with a pioneering work by *Greninger and colleagues* [6, 35], which has forecast such convergent evolutionary strategies of kobuviruses and enteroviruses based on extensive mutagenesis of the ACBD3-3A interface. There are still several other picornavirus genera proposed to recruit ACBD3 through the 3A: GOLD interaction (such as salivirus, hepatovirus, or parechovirus [6]) whose 3A: GOLD complexes remain structurally unexplored. Structural details of their ACBD3 recruitment uncovering whether they utilize the mechanism described for enteroviruses, kobuviruses, or another mechanism distinct from those two, remain to be further elucidated.

## Materials and methods

### Plasmids

For expression in *E*. *coli*, full-length human ACBD3 and various enterovirus 3A proteins and their deletion mutants were cloned into pRSFD vector (Novagen) with an *N*-terminal 6xHis tag followed by a GB1 solubility tag and a TEV protease cleavage site using PCR and restriction cloning. For bacterial expression of the EGFP-fusion proteins, the EGFP encoding sequence was inserted between the TEV cleavage site and the target gene encoding regions. For expression of the EGFP-fusion proteins in human cells, target genes encoding regions were recloned into pEGFP-C1 vector (Clontech) with an *N*-terminal EGFP tag. For expression of the GST-, mAmetrine-, and mPlum-fusion proteins in human cells, the EGFP encoding region was replaced by GST or corresponding fluorescent protein encoding sequence by PCR and restriction cloning. The pRib-EVD68/mCherry plasmid for viral subgenomic replicon assays was generated by subcloning of the EVD68 cDNA of a prototypical Fermon strain under T7 promoter and replacing the capsid proteins-encoding region with the mCherry fluorescent protein-encoding gene by Gibson assembly. Mutations were generated using the Q5 Site-Directed Mutagenesis Kit (New England BioLabs). All DNA constructs were verified by sequencing. The pEGFP-ACBD3 and pBIND-ACBD3 plasmids were kindly provided by Carolyn Machamer and Jun Sasaki, respectively. The mAmetrine-C1 and mPlum-C1 plasmids were gifts from Robert Campbell and Michael Davidson (Addgene plasmids #54660 [36] and #54839 [37]). The pEGFP-GalT plasmid was a gift from Jennifer Lippincott-Schwartz (Addgene plasmid #11929 [38]).

### Protein expression and purification

All recombinant proteins used in this study were bacterially expressed as fusion proteins with an *N*-terminal 6xhistidine (His_6_) tag followed by a GB1 solubility tag and a TEV protease cleavage site. For the crystallographic analysis of the GOLD: 3A complexes, the *N*-terminally His_6_-GB1-TEV site-fused cytoplasmic domains of the 3A proteins were directly co-expressed with the untagged ACBD3 GOLD domain. The proteins were expressed in *E*. *coli* BL21 DE3 NiCo cells (New England Biolabs) using the autoinduction ZY medium. Bacterial cells were harvested and lysed in the lysis buffer (50 mM Tris pH 8, 300 mM NaCl, 3 mM β-mercaptoethanol, 30 mM imidazole, 10% glycerol), the lysate was incubated with the HisPur Ni-NTA Superflow agarose (Thermo Fisher Scientific), and the bound proteins were extensively washed with the wash buffer (50 mM Tris pH 8, 300 mM NaCl, 1 mM β-mercaptoethanol, 20 mM imidazole). The protein was eluted with the elution buffer (50 mM Tris pH 8, 200 mM NaCl, 3 mM β-mercaptoethanol, 300 mM imidazole).

For the biochemical analysis of the 3A proteins by microscale thermophoresis or SAXS, the *N*-terminal His_6_-GB1 tags were preserved uncleaved to increase protein solubility and to avoid aggregation at required concentrations. For the crystallographic analysis of the GOLD: 3A complexes and for the biochemical analysis of the GOLD-3A fusion proteins by SAXS, the *N*-terminal His_6_-GB1 tags were removed with home-made TEV protease. Next, the proteins were purified using the size exclusion chromatography at HiLoad 16/60 Superdex 75 or Superdex 200 prep grade columns (GE Healthcare) in the storage buffer (10 mM Tris pH 8, 200 mM NaCl, 3 mM β-mercaptoethanol). In addition, the GOLD: 3A complexes used for the crystallographic analysis were further purified by reverse immobilized metal affinity chromatography using the HisTrap HP column (GE Healthcare), while the EGFP-fused ACBD3 GOLD domain used for microscale thermophoresis was further purified using the ion exchange chromatography at a MonoQ 10/100 GL column (GE Healthcare) and then dialyzed back into the storage buffer. The molecular weight and purity of all proteins was verified by SDS-PAGE (S9 Fig) and Matrix-Assisted Laser Desorption/Ionisation (MALDI). Purified proteins were concentrated to 1–10 mg/ml, aliquoted, flash frozen in the liquid nitrogen, and stored at -80 °C until needed.

### Crystallization and crystallographic analysis

Crystals grew at 291 K in sitting drops by the vapor diffusion method. They were cryoprotected, flash frozen in liquid nitrogen, and analyzed. Measurements were carried out at the MX14.1 beamline of the synchrotron BESSY II at Helmholtz-Zentrum Berlin [39]. The crystallographic datasets were collected from single frozen crystals. Data were integrated and scaled using *XDS* [40] and *XDSAPP* [41]. Structures were solved by molecular replacement using the uncomplexed ACBD3 GOLD domain structure (pdb code 5LZ1) as a search model. The initial models were obtained with *Phaser* [42] from the *Phenix* package [43]. The models were further improved using automatic model building with *Buccaneer* [44] from the *CCP4* suite [45], automatic model refinement with *Phenix*.*refine* [46] from the *Phenix* package [43], and manual model building with *Coot* [47]. Statistics for data collection and processing, structure solution and refinement are summarized in Table 1. Structural figures were generated with *PyMol* [48]. The atomic coordinates and structural factors were deposited in the Protein Data Bank (www.pdb.org).

### Microscale thermophoresis (MST)

MST measurements were carried out using the Monolith NT.115 instrument (NanoTemper Technologies) according to the manufacturer's instructions. The Monolith NT.115 standard treated capillaries were loaded with a mixture of a recombinant EGFP-fused protein at a constant concentration of 150 nM in the MST buffer (30 mM Tris pH 7.4, 150 mM NaCl, 3 mM β-mercaptoethanol) and its binding partner in the appropriate series of concentrations. The thermophoretic motion of the fluorescent protein and its temperature-dependent changes of fluorescence were analyzed with the Monolith NT Analysis Software.

### Tissue cultures and transfections

Human cervical-carcinoma cells HeLa (American Type Culture Collection / ATCC), embryonic kidney cells HEK293T (ATCC), and keratinocytes HaCaT (AddexBio) were maintained in Dulbecco's modified Eagle's medium (Sigma) supplemented with 10% fetal calf serum (Gibco). Human glioblastoma cells U-87 MG (ATCC) were maintained in Minimum Essential Medium Eagle (Sigma) supplemented with 10% fetal calf serum (Gibco), GlutaMAX Supplement (Thermo Fisher Scientific), and non-essential amino acids (Biowest). HeLa cells were transfected using Lipofectamine2000 reagent (Thermo Fisher Scientific) or X-tremeGENE HP DNA Transfection reagent (Sigma/Roche) according to manufacturer's instructions. Transfections of HEK293T cells were performed using polyethylenimine (Sigma) or Fugene6 (Promega).

### Co-immunoprecipitation assay

HEK293T cells were transfected with the appropriate mutants of the EGFP-fused EVD68 3A protein and GST-fused ACBD3. The next day, cells were harvested, washed twice with phosphate-buffered saline (PBS) and lysed in the ice-cold lysis buffer (20 mM Tris pH 7.4, 100 mM NaCl, 50 mM NaF, 10 mM EDTA, 10% glycerol, 1% NP-40), supplemented with protease inhibitors (Complete protease inhibitor cocktail, Sigma/Roche). After solubilizing for 15 min on ice, the lysate was pre-cleared by centrifugation at 16,000g for 15 min. The resulting supernatant was incubated with sepharose beads coupled either to GFP nanobody (GFP-Trap, ChromoTek) or glutathione (Protino Glutathione Agarose, Macherey-Nagel) for 1h at 4 °C. After three washes with 10 volumes of the lysis buffer, the bound proteins were directly eluted with the Laemmli sample buffer, subjected to SDS-PAGE, and analyzed by immunoblotting. The whole cell lysates and eluted proteins were stained with mouse monoclonal antibodies to ACBD3 (Santa Cruz Biotechnology, sc-101277) and GFP (Santa Cruz Biotechnology, sc-9996). The images were acquired using the LI-COR Odyssey Infrared Imaging System.

### Förster resonance energy transfer (FRET) assay

HeLa cells were co-transfected with plasmids encoding target proteins fused to the FRET pair of fluorescent proteins mAmetrine ("donor") and mPlum ("acceptor"). The next day, cells were harvested, washed twice with PBS, and analyzed by flow cytometry using BD LSR Fortessa (BD Biosciences). The donor and acceptor fluorescence as well as the energy transfer was determined using the optical configurations as follows: mAmetrine—405 nm laser—525/50 nm bandpass filter; mPlum—561 nm laser—670/30 nm bandpass filter; FRET—405 nm laser—655/8 nm bandpass filter. Acquired data were analyzed with the FlowJo software. The acquired fluorescence intensities were compensated and the same gate corresponding to the live transfected cells with the approximately 1:1 donor:acceptor ratio was applied to all samples. The acquired events were binned on the basis of the acceptor fluorescence intensity, and the average FRET fluorescence intensities of each bin were plotted against the respective acceptor fluorescence intensity.

### Mammalian two-hybrid assay

HEK293T cells grown in 96-well plates were co-transfected with 50 ng of each pACT, pBIND, and pG5Luc plasmids using Fugene6 (Promega). At 24 hours post transfection, the cells were lysed, and both firefly and *Renilla* luciferase activities were measured using the Dual-Luciferase assay kit (Promega) and Centro LB 960 luminometer (Berthold Technologies) according to the manufacturer's instructions. The firefly luciferase activity was normalized to the *Renilla* luciferase activity (used as an internal control of the transfection efficiency) and then to the activity determined in cells co-expressing wild-type ACBD3 and 3A (which was set to 100%).

### Immunofluorescence assay

HeLa cells grown on coverslips in 24-well plates were transfected with 400 ng of the plasmid DNA using Lipofectamine2000 (Thermo Fisher Scientific). At 16 hours post transfection, the cells were fixed with 4% paraformaldehyde for 15 min at room temperature, permeabilized with 0.1% Triton X-100 in PBS for 5 min, and immunostained with the appropriate primary and secondary antibodies diluted in 2% normal goat serum in PBS. Sources of the antibodies were as follows: anti-ACBD3 (Sigma, WH0064746M1), anti-PI4KB (Merck, 06–578), anti-GM130 (BD Biosciences, 610822), anti-giantin (Enzo Life Science, ALX-804-600-C100), anti-myc (Thermo Fisher Scientific, PA1-981), anti-PI4P (Echelon, Z-P004), and goat-anti-mouse and goat-anti-rabbit secondary antibodies conjugated to AlexaFluor 488, 596, or 647 (Molecular Probes). Nuclei were stained with DAPI. Coverslips were mounted with FluorSave (Calbiochem), and confocal imaging was performed with a Leica SpeII confocal microscope.

### Virus subgenomic replicon assay

The pRib-EVD68/mCherry wild-type and mutant plasmids were linearized by cleavage with SalI-HF (Thermo Fisher Scientific) and purified using the mini spin columns (Epoch Life Science). Viral subgenomic replicon RNA was generated with TranscriptAid T7 High Yield Transcription Kit (Thermo Fisher Scientific) and purified using the RNeasy mini spin columns (Qiagen). For replicon assays, U-87 MG or HaCaT cells grown in 12-well plates were transfected with T7-amplified RNA using the TransIT mRNA transfection kit (Mirus Bio). At 12 hours post transfection, the reporter mCherry fluorescence was determined by flow cytometry using BD LSR Fortessa (BD Biosciences) and the following optical configuration: 561 nm laser, 670/30 nm bandpass filter. Acquired data were analyzed with the FlowJo software. The level of RNA replication was expressed as a transfection efficiency-normalized percentage of cells with the mCherry signal above the threshold determined using the viral polymerase-lacking mutant Δ3D^pol^.

To test the effect of PI4KB inhibition on virus replication, a PI4KB-specific inhibitor (compound 10 from [10] kindly provided by Radim Nencka) was added to the medium at a final concentration of 1 μM 30 min prior transfection of the viral subgenomic replicon RNA.

### Virus replication rescue assay

Wild-type or ACBD3 knock-out HeLa cells grown in 96-well plates were transfected with plasmids encoding wild-type or mutant ACBD3 or another Golgi-resident protein GalT as a control. At 24 hours post transfection, the cells were infected with the *Renilla* luciferase-expressing CVB3 virus (RLucCVB3) [49]. At 8 hours post infection, the intracellular *Renilla* luciferase activity was determined using the *Renilla* luciferase assay system (Promega) and a Centro LB 960 luminometer (Berthold Technologies). The *Renilla* luciferase activity was normalized to the activity determined in wild-type ACBD3-expressing cells (which was set to 100%).

### Small angle X-ray scattering (SAXS) measurements and data analysis

Proteins were dialyzed against the SAXS buffer (30 mM Tris pH 7.4, 150 mM NaCl, 1 mM TCEP) and concentrated as follows: c_1_ = 1.03 mg/ml, c_2_ = 1.4 mg/ml and c_3_ = 1.54 mg/ml for the wild-type GOLD-3A fusion protein, and c_1_ = 0.96 mg/ml, c_2_ = 2.36 mg/ml and c_3_ = 3.26 mg/ml for the GOLD-3A LVVY mutant. The SAXS data were collected using the beamlines BioSAXS Beamline BM29 (ESRF, Grenoble) and EMBL SAXS beamline P12 (Petra III DESY, Hamburg) that are both equipped with the 2M Pilatus detector (Dectris). The three datasets overlay after rescaling, indicating no protein aggregation in the samples. To structurally interpret the SAXS data, we incorporated the missing loop (D437-K473) into the structures of the wild-type GOLD-3A dimer and GOLD-3A LVVY mutant monomer. Next, we performed the coarse-grained molecular simulations [50] in which only the conformations of the D437-K473 loop were sampled while the crystallized portion of the protein was kept rigid, yielding 10,000 structural models of both proteins. For all structural models, we computed the SAXS intensity profiles using the previously developed algorithms [51], compared them individually to the experimental SAXS data, and selected the models best fitting the SAXS data collected on the samples with the highest protein concentrations. The best models fit the SAXS data with χ = 1.5 for the wild-type GOLD-3A dimer and χ = 1.4 for the GOLD-3A LVVY mutant monomer.

### Molecular modeling and molecular dynamics (MD) simulations

The intrinsically disordered region of the ACBD3 GOLD domain, which was missing in the crystal structure (D437-K473), was modeled as described previously [23]. The *C*-terminal segment of the poliovirus 3A protein (N57-Q87) was modeled as a loop (N57-M60) followed by a transmembrane alpha helix (T61-F83) and a short *C*-terminal tail (A84-Q87). This segment was positioned in a planar segment of a lipid bilayer using the PPM server [52].

MD simulations of the ACBD3 GOLD domain in complex with the poliovirus 3A protein in the environment of a lipid bilayer were performed following the procedures recently used to study the ACBD3 GOLD domain in complex with the aichivirus 3A protein [23]. The initial system for MD simulations was prepared using VMD version 1.9.2 [53]. Namely, a POPC bilayer segment with the lateral dimensions of 10 nm by 10 nm was formed using the Membrane Plugin version 1.1 in VMD. The GOLD: 3A complex was placed on top of the resulting lipid patch. The lipids overlapping with the transmembrane alpha helix of the 3A protein were removed. The system was solvated using the Solvate Plugin version 1.5 in VMD. Sodium and chloride ions were added to neutralize the simulated system and to reach a physiological concentration of 150 mM. The MD simulations were performed using the NAMD package [54] version 2.9. The CHARMM22 force field [55, 56] with the CMAP correction [57] and the TIP3P water model were used. The simulations were carried out in the NPT ensemble. Temperature was kept at 298K through a Langevin thermostat with a damping coefficient of 1/ps. Pressure was maintained at 1 atm using the Langevin piston Nose-Hoover method with a damping timescale of 50 fs and an oscillation timescale of 100 fs. Short-range non-bonded interactions were cut off smoothly between 1 and 1.2 nm. Long-range electrostatic interactions were computed using the particle-mash Ewald method with a grid spacing of 0.1 nm. Simulations were performed with an integration time step of 2 fs. After initial energy minimization with a conjugate gradient method, a 10 ns simulation was performed with constraints on the protein backbone atoms in order to equilibrate the lipids, ions and water molecules. Namely, a harmonic potential with the spring constant of 5 kcal/(mol Å^2^) was applied to all backbone atoms of the GOLD: 3A complex. After the equilibration, the system was simulated with no constrains for 200 ns. The resulting MD trajectory was visualized and analyzed using VMD.

To localize the PI4KB/OSBP-inhibition resistance-conferring mutations within the structure of the GOLD: CVB3 3A complex, a homology model of this complex was generated by the I-TASSER server [58] using the crystal structure of the GOLD: EVD68 3A complex as a template.

### Statistical analysis

In the graphs, data are presented as mean values ± standard errors of the means (SEMs) based on three independent experiments, unless stated otherwise. For statistical analyses, two-tailed two-sample Student's t-tests were used. P-values below 0.05 were considered significant.

### Accession numbers

The crystal structures of the ACBD3 GOLD domain in complex with the 3A proteins from EVD68, RVB14, and PV1 (L24A mutant), and the ACBD3 GOLD domain fused to the 3A proteins from EVA71, EVD68 (wild type), and EVD68 (LVVY mutant) from this publication have been submitted to the Protein Data Bank (www.pdb.org) and assigned the identifiers 6HLN, 6HLT, 6HLV, 6HLW, 6HM8, and 6HMV, respectively.

## Supporting information

S1 Fig Design of the 3A and ACBD3 mutants used in this study. **a**, Changes of the ACBD3—3A interaction energies of to-alanine mutants of 3A as obtained with the *Pssm* tool of the *FoldX* software package [24] using the crystal structures presented in this work. Amino acid residues used for further design of single and multiple 3A mutants are marked by asterisks. **b-c**, Changes of the ACBD3—3A interaction energies of to-alanine mutants of ACBD3 within the regions V368-T436 (**b**) and P474-R528 (**c**) calculated and visualized as in (**a**). Data for the intrinsically disordered region D437-K473 are not available. The ACBD3 E419A mutant was released from the Golgi (S4 Fig, panel e) and, therefore, excluded from further design of multiple mutants.
(TIF)

S2 Fig PI4P levels in the wild-type and mutant EVD68 3A-expressing cells. EGFP-fused wild-type EVD68 3A and its mutants were overexpressed in HeLa cells. The cells were fixed and immunostained with the anti-PI4P antibody (Echelon #Z-P004). Scale bars represent 10 μm.
(TIF)

S3 Fig Analysis of the EVD68 mutants by the subgenomic replicon assay (raw data). **a-b**, Human glioblastoma cells U-87 MG (**a**) or keratinocytes HaCaT (**b**) were transfected with the T7-amplified EVD68 Fermon strain subgenomic replicon wild-type RNA or its mutants as indicated, and the reporter mCherry fluorescence was determined by flow cytometry. Staining with the Hoechst33258 dye was added to determine the cell viability. The level of RNA replication was expressed as a percentage of cells with the mCherry signal above the threshold determined using the viral polymerase-lacking mutant Δ3D^pol^ (red region), further normalized to the transfection efficiency (black region). Data from one representative experiment are shown; please see Figs 3f, 3g and 5i for the quantification based on two independent experiments. PI4KBi, a PI4KB-specific inhibitor (compound 10 in *Mejdrova et al*. [10]).
(TIF)

S4 Fig Analysis of the ACBD3-3A interface. **a**, **e**, Localization of the ACBD3 mutants. EGFP-fused wild-type ACBD3 or its mutants were overexpressed in HeLa ACBD3 knock-out cells. Cells were fixed and immunostained with the anti-GM130 antibody (marker of Golgi). Scale bars represent 10 μm. **b**, **d**, **f**, Rescue of enterovirus replication by the ACBD3 mutants. HeLa ACBD3 knock-out cells were transfected with wild-type ACBD3 or its mutants, and enterovirus replication was determined using the *Renilla* luciferase-expressing CVB3 virus by the *Renilla* luciferase assay system. GalT and ACBD3 F258A/Q259A were used as controls. **c**, Mammalian-two-hybrid assay with the ACBD3 mutants and wild-type 3A. HeLa cells were transfected as indicated and the firefly luciferase activity normalized to the *Renilla* luciferase activity was determined using a dual-luciferase reporter assay system.
(TIF)

S5 Fig Design and crystallization of the GOLD-3A fusion protein and its LVVY mutant. **a**, Changes of the dimerization energies of to-alanine mutants of the GOLD: 3A complexes as obtained with the *Pssm* tool of the *FoldX* software package [24] using the crystal structures presented in this work. Residues forming the hydrophobic core of the dimerization interface are marked by asterisks. In the inset, a detailed view of the EVD68 3A dimerization interface colored as in Fig 5a is shown. **b**, Overall fold of the GOLD: EVD68 3A complex formed by two individual proteins (left), wild-type GOLD—EVD68 3A fusion protein (middle), and its LVVY mutant (right). The ACBD3 GOLD domain is depicted in grey, the EVD68 3A protein in rainbow colors from blue (*N* terminus) to red (*C* terminus). Residues forming the hydrophobic core of the dimerization interface (mutated in the LVVY mutant) are marked by asterisks. **c**, Statistics for data collection and processing, structure solution and refinement of the proteins and protein complexes shown in (**b**). Numbers in parentheses refer to the highest resolution shell of the respective dataset.
(TIF)

S6 Fig Analysis of the dimerization of selected EVD68 3A mutants. In the upper panels, a detailed view of the EVD68 3A dimerization interface colored as in Fig 5a is shown, except for the mutated residues, which are depicted in black. Distances of the closest atom pairs of selected residues are shown in Angstroms. Homology models of the mutant 3A proteins were generated by mutating the respective residues in Coot followed by the energy minimization in Swiss-PDBViewer. In the lower panels, elution profiles of the GB1-fused wild-type and mutant EVD68 3A proteins are shown. Each protein at a final concentration of 100 μM was analyzed by size exclusion chromatography using the Superdex 10/300 Increase column (GE Healthcare) and its elution was monitored by the absorbance at 280 nm.
(TIF)

S7 Fig Analysis of the dimerization interface of the GOLD-3A complexes. **a**, Structural model of the dimer of the GOLD—EVD68 3A fusion proteins used for the SAXS analysis. The ACBD3 GOLD domains are shown in cartoon representation with a semi-transparent surface and colored in grey except for the modeled intrinsically disordered loops of ACBD3 (D437-K473), which are depicted in blue. The EVD68 3A proteins are colored in yellow and red. **b-c**, Crystal packing of the wild-type GOLD—EVD68 3A fusion protein (**b**) and its LVVY mutant (**c**). The content of two unit cells with protein backbones in cartoon representation is shown. The ACBD3 GOLD domain is depicted in grey, the EVD68 3A protein in red. Wild-type 3A forms a crystal-packing contact through the 3A dimerization interface (**b**), while in the case of the LVVY mutant this contact is not preserved and instead, the *N*-terminal alpha helix of 3A forms a crystal-packing contact with the *C*-terminal beta strand of another 3A molecule (**c**).
(TIF)

S8 Fig Localization of the PI4KB/OSBP-inhibition resistance-conferring mutations. **a**, Localization of the PI4KB/OSBP-inhibition resistance-conferring mutations within the primary sequences of the enterovirus 3A proteins. Sequences of the 3A proteins of selected enteroviruses used in this study were aligned as in Fig 1a. Secondary structures present in the crystal structures of the ACBD3: 3A complexes (colored in light blue) and the hydrophobic alpha helix anchoring the 3A proteins to the membrane (colored in red) are indicated above the sequences. ACBD3-binding regions are shaded in grey. Residues whose mutations have been reported to confer resistance to the PI4KB/OSBP-specific inhibitors, i.e. PV1 N45Y, R54W, N57D, A70T, and A71S, CVB3 V45A, I54F, and H57Y, and RVB14 E30D/V/Q, I42V, and M54I [31–34], are highlighted in red. **b**, Localization of the PI4KB/OSBP-inhibition resistance-conferring mutations within the structures of the GOLD: 3A complexes. A homology model of the GOLD: CVB3 3A complex was generated by the I-TASSER server [58] using the crystal structure of the GOLD: EVD68 3A complex as a template. The ACBD3 GOLD domain is shown in cartoon representation with a semi-transparent surface and colored in grey; the enterovirus 3A proteins are depicted in yellow. Residues whose mutations have been reported to confer resistance to the PI4KB/OSBP-specific inhibitors are highlighted in red.
(TIF)

S9 Fig Recombinant proteins used in the study. Recombinant proteins used for the crystallographic analysis (**a**), microscale thermophoresis (**b**), and SAXS analysis (**c**) were resolved by SDS-PAGE using the 15% polyacrylamide gels and stained with Coomassie Blue. The hyphen signs ("-") indicate fusion proteins, while the plus signs ("+") indicate complexes of two individual proteins. The uncomplexed viral proteins used for microscale thermophoresis and SAXS analysis were fused to the B1 domain of streptococcal protein G ("GB1 tag") to improve their solubility and to avoid their non-specific aggregation. Asterisks indicate the successfully crystallized proteins and protein complexes.
(TIF)

## References

[1] Tapparel C, Siegrist F, Petty TJ, Kaiser L. Picornavirus and enterovirus diversity with associated human diseases. Infect Genet Evol. 2013;14:282–93. 10.1016/j.meegid.2012.10.016 23201849

[2] Wessels E, Duijsings D, Niu T-K, Neumann S, Oorschot VM, de Lange F, et al A viral protein that blocks Arf1-mediated COP-I assembly by inhibiting the guanine nucleotide exchange factor GBF1. Dev Cell. 2006;11:191–201. 10.1016/j.devcel.2006.06.005 16890159

[3] Greninger AL, Knudsen GM, Betegon M, Burlingame AL, DeRisi JL. The 3A protein from multiple picornaviruses utilizes the golgi adaptor protein ACBD3 to recruit PI4KIIIβ. J Virol. 2012;86:3605–16. 10.1128/JVI.06778-11 22258260 PMC3302542

[4] Liao J, Guan Y, Chen W, Shi C, Yao D, Wang F, et al ACBD3 is required for FAPP2 transferring glucosylceramide through maintaining the Golgi integrity. J Mol Cell Biol. 2018 10.1093/jmcb/mjy030 29750412 PMC6734144

[5] Sohda M, Misumi Y, Yamamoto A, Yano A, Nakamura N, Ikehara Y. Identification and characterization of a novel Golgi protein, GCP60, that interacts with the integral membrane protein giantin. J Biol Chem. 2001;276:45298–306. 10.1074/jbc.M108961200 11590181

[6] Greninger AL, Knudsen GM, Betegon M, Burlingame AL, DeRisi JL. ACBD3 interaction with TBC1 domain 22 protein is differentially affected by enteroviral and kobuviral 3A protein binding. mBio. 2013;4:e00098–13. 10.1128/mBio.00098-13 23572552 PMC3622926

[7] Klima M, Tóth DJ, Hexnerova R, Baumlova A, Chalupska D, Tykvart J, et al Structural insights and in vitro reconstitution of membrane targeting and activation of human PI4KB by the ACBD3 protein. Sci Rep. 2016;6:23641 10.1038/srep23641 27009356 PMC4806292

[8] Lei X, Xiao X, Zhang Z, Ma Y, Qi J, Wu C, et al The Golgi protein ACBD3 facilitates Enterovirus 71 replication by interacting with 3A. Sci Rep. 2017;7:44592 10.1038/srep44592 28303920 PMC5356004

[9] Hsu N-Y, Ilnytska O, Belov G, Santiana M, Chen Y-H, Takvorian PM, et al Viral reorganization of the secretory pathway generates distinct organelles for RNA replication. Cell. 2010;141:799–811. 10.1016/j.cell.2010.03.050 20510927 PMC2982146

[10] Mejdrova I, Chalupska D, Plackova P, Mueller C, Sala M, Klima M, et al Rational design of novel highly potent and selective phosphatidylinositol 4-kinase III beta (PI4KB) inhibitors as broad-spectrum antiviral agents and tools for chemical biology. J Med Chem. 2017;60(1):100–18. 10.1021/acs.jmedchem.6b01465 28004945

[11] Xiao X, Lei X, Zhang Z, Ma Y, Qi J, Wu C, et al Enterovirus 3A facilitates viral replication by promoting PI4KB-ACBD3 interaction. J Virol. 2017 10.1128/JVI.00791-17 28701404 PMC5599760

[12] Arita M. Phosphatidylinositol-4 kinase III beta and oxysterol-binding protein accumulate unesterified cholesterol on poliovirus-induced membrane structure. Microbiol Immunol. 2014;58:239–56. 10.1111/1348-0421.12144 24527995

[13] Banerjee S, Aponte-Diaz D, Yeager C, Sharma SD, Ning G, Oh HS, et al Hijacking of multiple phospholipid biosynthetic pathways and induction of membrane biogenesis by a picornaviral 3CD protein. PLoS Pathog. 2018;14(5):e1007086 10.1371/journal.ppat.1007086 29782554 PMC5983871

[14] Sasaki J, Ishikawa K, Arita M, Taniguchi K. ACBD3-mediated recruitment of PI4KB to picornavirus RNA replication sites. EMBO J. 2012;31:754–66. 10.1038/emboj.2011.429 22124328 PMC3273392

[15] Ishikawa-Sasaki K, Sasaki J, Taniguchi K. A complex comprising phosphatidylinositol 4-kinase IIIβ, ACBD3, and Aichi virus proteins enhances phosphatidylinositol 4-phosphate synthesis and is critical for formation of the viral replication complex. J Virol. 2014;88:6586–98. 10.1128/JVI.00208-14 24672044 PMC4054359

[16] McPhail JA, Ottosen EH, Jenkins ML, Burke JE. The molecular basis of Aichi virus 3A protein activation of phosphatidylinositol 4 kinase IIIbeta, PI4KB, through ACBD3. Structure. 2017;25(1):121–31. 10.1016/j.str.2016.11.016 27989622

[17] Dubankova A, Humpolickova J, Klima M, Boura E. Negative charge and membrane-tethered viral 3B cooperate to recruit viral RNA dependent RNA polymerase 3Dpol. Sci Rep. 2017;7(1):17309 10.1038/s41598-017-17621-6 29230036 PMC5725453

[18] Mesmin B, Bigay J, Moser von Filseck J, Lacas-Gervais S, Drin G, Antonny B. A four-step cycle driven by PI(4)P hydrolysis directs sterol/PI(4)P exchange by the ER-Golgi tether OSBP. Cell. 2013;155:830–43. 10.1016/j.cell.2013.09.056 24209621

[19] Chung J, Torta F, Masai K, Lucast L, Czapla H, Tanner LB, et al PI4P/phosphatidylserine countertransport at ORP5- and ORP8-mediated ER-plasma membrane contacts. Science. 2015;349:428–32. 10.1126/science.aab1370 26206935 PMC4638224

[20] Roulin PS, Lötzerich M, Torta F, Tanner LB, van Kuppeveld FJM, Wenk MR, et al Rhinovirus uses a phosphatidylinositol 4-phosphate/cholesterol counter-current for the formation of replication compartments at the ER-Golgi interface. Cell Host Microbe. 2014;16:677–90. 10.1016/j.chom.2014.10.003 25525797

[21] Lyoo H, van der Schaar HM, Dorobantu CM, Rabouw HH, Strating JRPM, van Kuppeveld FJM. ACBD3 is an essential pan-enterovirus host factor that mediates the interaction between viral 3A protein and cellular protein PI4KB. mBio. 2019;10(1):e02742–18. 10.1128/mBio.02742-18 30755512 PMC6372799

[22] Strauss DM, Glustrom LW, Wuttke DS. Towards an understanding of the poliovirus replication complex: the solution structure of the soluble domain of the poliovirus 3A protein. J Mol Biol. 2003;330:225–34. 10.1016/s0022-2836(03)00577-1 12823963

[23] Klima M, Chalupska D, Rozycki B, Humpolickova J, Rezabkova L, Silhan J, et al Kobuviral non-structural 3A proteins act as molecular harnesses to hijack the host ACBD3 protein. Structure. 2017;25(2):219–30. 10.1016/j.str.2016.11.021 28065508

[24] Schymkowitz J, Borg J, Stricher F, Nys R, Rousseau F, Serrano L. The FoldX web server: an online force field. Nucleic Acids Res. 2005;33:W382–8. 10.1093/nar/gki387 15980494 PMC1160148

[25] Wessels E, Notebaart RA, Duijsings D, Lanke K, Vergeer B, Melchers WJ, et al Structure-function analysis of the coxsackievirus protein 3A: identification of residues important for dimerization, viral RNA replication, and transport inhibition. J Biol Chem. 2006;281(38):28232–43. 10.1074/jbc.M601122200 16867984

[26] Dorobantu CM, van der Schaar HM, Ford LA, Strating JRPM, Ulferts R, Fang Y, et al Recruitment of PI4KIIIβ to coxsackievirus B3 replication organelles is independent of ACBD3, GBF1, and Arf1. J Virol. 2014;88:2725–36. 10.1128/JVI.03650-13 24352456 PMC3958084

[27] Dorobantu CM, Ford-Siltz LA, Sittig SP, Lanke KHW, Belov GA, van Kuppeveld FJM, et al GBF1- and ACBD3-independent recruitment of PI4KIIIβ to replication sites by rhinovirus 3A proteins. J Virol. 2015;89:1913–8. 10.1128/JVI.02830-14 25410869 PMC4300732

[28] Esser-Nobis K, Harak C, Schult P, Kusov Y, Lohmann V. Novel perspectives for hepatitis A virus therapy revealed by comparative analysis of hepatitis C virus and hepatitis A virus RNA replication. Hepatology. 2015;62(2):397–408. 10.1002/hep.27847 25866017 PMC7165973

[29] Berryman S, Moffat K, Harak C, Lohmann V, Jackson T. Foot-and-mouth disease virus replicates independently of phosphatidylinositol 4-phosphate and type III phosphatidylinositol 4-kinases. J Gen Virol. 2016;97(8):1841–52. 10.1099/jgv.0.000485 27093462 PMC5156328

[30] Dorobantu CM, Albulescu L, Harak C, Feng Q, van Kampen M, Strating JRPM, et al Modulation of the host lipid landscape to promote RNA virus replication: the picornavirus encephalomyocarditis virus converges on the pathway used by hepatitis C virus. PLoS Pathog. 2015;11:e1005185 10.1371/journal.ppat.1005185 26406250 PMC4583462

[31] Heinz BA, Vance LM. The antiviral compound enviroxime targets the 3A coding region of rhinovirus and poliovirus. J Virol. 1995;69(7):4189–97. 7769678 10.1128/jvi.69.7.4189-4197.1995 PMC189156

[32] De Palma AM, Thibaut HJ, van der Linden L, Lanke K, Heggermont W, Ireland S, et al Mutations in the nonstructural protein 3A confer resistance to the novel enterovirus replication inhibitor TTP-8307. Antimicrob Agents Chemother. 2009;53(5):1850–7. 10.1128/AAC.00934-08 19237651 PMC2681499

[33] Arita M, Wakita T, Shimizu H. Cellular kinase inhibitors that suppress enterovirus replication have a conserved target in viral protein 3A similar to that of enviroxime. J Gen Virol. 2009;90(Pt 8):1869–79. 10.1099/vir.0.012096-0 19439558

[34] Arita M, Bigay J. Poliovirus evolution towards independence from the phosphatidylinositol-4 kinase III beta/oxysterol-binding protein family I pathway. ACS Infect Dis. 2019 10.1021/acsinfecdis.9b00038 30919621

[35] Greninger AL. Picornavirus—host interactions to construct viral secretory membranes. Prog Mol Biol Transl. 2015;129:189–212. 10.1016/bs.pmbts.2014.10.007 25595805 PMC12716941

[36] Ai HW, Hazelwood KL, Davidson MW, Campbell RE. Fluorescent protein FRET pairs for ratiometric imaging of dual biosensors. Nat Methods. 2008;5(5):401–3. 10.1038/nmeth.1207 18425137

[37] Kremers GJ, Hazelwood KL, Murphy CS, Davidson MW, Piston DW. Photoconversion in orange and red fluorescent proteins. Nat Methods. 2009;6(5):355–8. 10.1038/nmeth.1319 19363494 PMC2675661

[38] Cole NB, Smith CL, Sciaky N, Terasaki M, Edidin M, Lippincott-Schwartz J. Diffusional mobility of Golgi proteins in membranes of living cells. Science. 1996;273(5276):797–801. 10.1126/science.273.5276.797 8670420

[39] Mueller U, Darowski N, Fuchs MR, Förster R, Hellmig M, Paithankar KS, et al Facilities for macromolecular crystallography at the Helmholtz-Zentrum Berlin. J Synchrotron Radiat. 2012;19:442–9. 10.1107/S0909049512006395 22514183 PMC3408958

[40] Kabsch W. XDS. Acta Crystallogr D. 2010;66:125–32. 10.1107/S0907444909047337 20124692 PMC2815665

[41] Krug M, Weiss MS, Heinemann U, Mueller U. XDSAPP: a graphical user interface for the convenient processing of diffraction data using XDS. J Appl Crystallogr. 2012;45:568–72. 10.1107/S0021889812011715

[42] McCoy AJ, Grosse-Kunstleve RW, Adams PD, Winn MD, Storoni LC, Read RJ. Phaser crystallographic software. J Appl Crystallogr. 2007;40:658–74. 10.1107/S0021889807021206 19461840 PMC2483472

[43] Adams PD, Afonine PV, Bunkóczi G, Chen VB, Davis IW, Echols N, et al PHENIX: a comprehensive Python-based system for macromolecular structure solution. Acta Crystallogr D. 2010;66:213–21. 10.1107/S0907444909052925 20124702 PMC2815670

[44] Cowtan K. The Buccaneer software for automated model building. 1. Tracing protein chains. Acta Crystallogr D. 2006;62:1002–11. 10.1107/S0907444906022116 16929101

[45] Winn MD, Ballard CC, Cowtan KD, Dodson EJ, Emsley P, Evans PR, et al Overview of the CCP4 suite and current developments. Acta Crystallogr D. 2011;67:235–42. 10.1107/S0907444910045749 21460441 PMC3069738

[46] Afonine PV, Grosse-Kunstleve RW, Echols N, Headd JJ, Moriarty NW, Mustyakimov M, et al Towards automated crystallographic structure refinement with phenix.refine. Acta Crystallogr D. 2012;68:352–67. 10.1107/S0907444912001308 22505256 PMC3322595

[47] Emsley P, Cowtan K. Coot: model-building tools for molecular graphics. Acta Crystallogr D. 2004;60:2126–32. 10.1107/S0907444904019158 15572765

[48] The PyMOL Molecular Graphics System, Version 1.8 Schrödinger, LLC.

[49] Lanke KH, van der Schaar HM, Belov GA, Feng Q, Duijsings D, Jackson CL, et al GBF1, a guanine nucleotide exchange factor for Arf, is crucial for coxsackievirus B3 RNA replication. J Virol. 2009;83(22):11940–9. 10.1128/JVI.01244-09 19740986 PMC2772713

[50] Kim YC, Hummer G. Coarse-grained models for simulations of multiprotein complexes: application to ubiquitin binding. J Mol Biol. 2008;375(5):1416–33. 10.1016/j.jmb.2007.11.063 18083189 PMC2343030

[51] Rozycki B, Kim YC, Hummer G. SAXS ensemble refinement of ESCRT-III CHMP3 conformational transitions. Structure. 2011;19(1):109–16. 10.1016/j.str.2010.10.006 21220121 PMC3032427

[52] Lomize MA, Lomize AL, Pogozheva ID, Mosberg HI. OPM: orientations of proteins in membranes database. Bioinformatics. 2006;22(5):623–5. 10.1093/bioinformatics/btk023 16397007

[53] Humphrey W, Dalke A, Schulten K. VMD: visual molecular dynamics. J Mol Graphics. 1996;14:33–8, 27–8. 10.1016/0263-7855(96)00018-5 8744570

[54] Phillips JC, Braun R, Wang W, Gumbart J, Tajkhorshid E, Villa E, et al Scalable molecular dynamics with NAMD. J Comput Chem. 2005;26:1781–802. 10.1002/jcc.20289 16222654 PMC2486339

[55] MacKerell AD, Bashford D, Bellott M, Dunbrack RL, Evanseck JD, Field MJ, et al All-atom empirical potential for molecular modeling and dynamics studies of proteins. J Phys Chem B. 1998;102:3586–616. 10.1021/jp973084f 24889800

[56] Klauda JB, Venable RM, Freites JA, O'Connor JW, Tobias DJ, Mondragon-Ramirez C, et al Update of the CHARMM all-atom additive force field for lipids: validation on six lipid types. J Phys Chem B. 2010;114:7830–43. 10.1021/jp101759q 20496934 PMC2922408

[57] MacKerell AD, Feig M, Brooks CL. Extending the treatment of backbone energetics in protein force fields: limitations of gas-phase quantum mechanics in reproducing protein conformational distributions in molecular dynamics simulations. J Comput Chem. 2004;25:1400–15. 10.1002/jcc.20065 15185334

[58] Yang J, Yan R, Roy A, Xu D, Poisson J, Zhang Y. The I-TASSER Suite: protein structure and function prediction. Nat Methods. 2015;12(1):7–8. 10.1038/nmeth.3213 25549265 PMC4428668

