# Supplementary figures and images for: Convergent evolution in the mechanisms of ACBD3 recruitment to picornavirus replication sites
Source: PLoS Pathog. 2019 Aug 5;15(8):e1007962. doi: 10.1371/journal.ppat.1007962 (PMC6695192; doi:10.1371/journal.ppat.1007962)

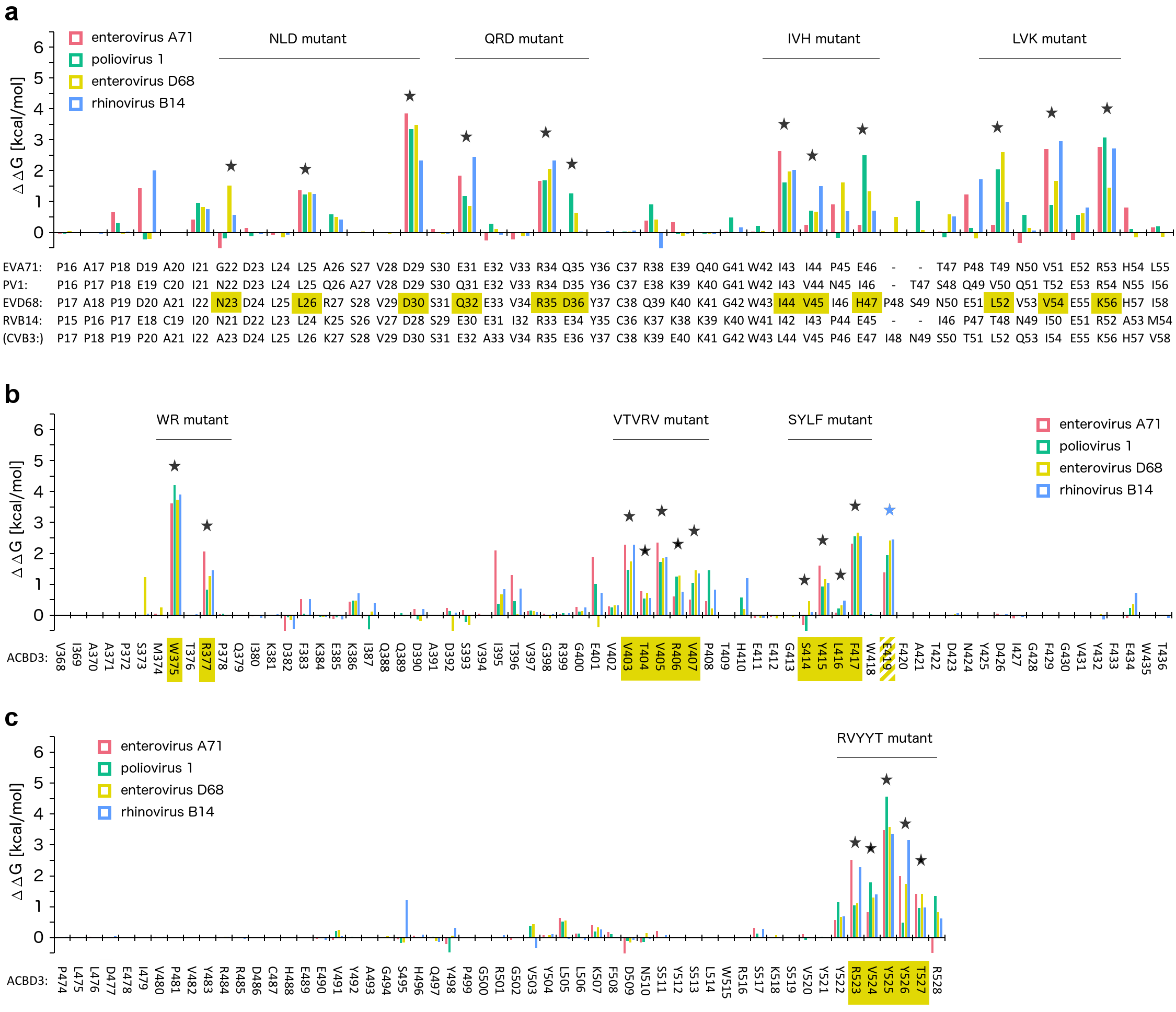

Supplement: S1 Fig — a, Changes of the ACBD3—3A interaction energies of to-alanine mutants of 3A as obtained with the Pssm tool of the FoldX software package [24] using the crystal structures presented in this work. Amino acid residues used for further design of single and multiple 3A mutants are marked by asterisks. b-c, Changes of the ACBD3—3A interaction energies of to-alanine mutants of ACBD3 within the regions V368-T436 (b) and P474-R528 (c) calculated and visualized as in (a). Data for the intrinsically disordered region D437-K473 are not available. The ACBD3 E419A mutant was released from the Golgi (S4 Fig, panel e) and, therefore, excluded from further design of multiple mutants. (TIF) [file ppat.1007962.s001.tif]

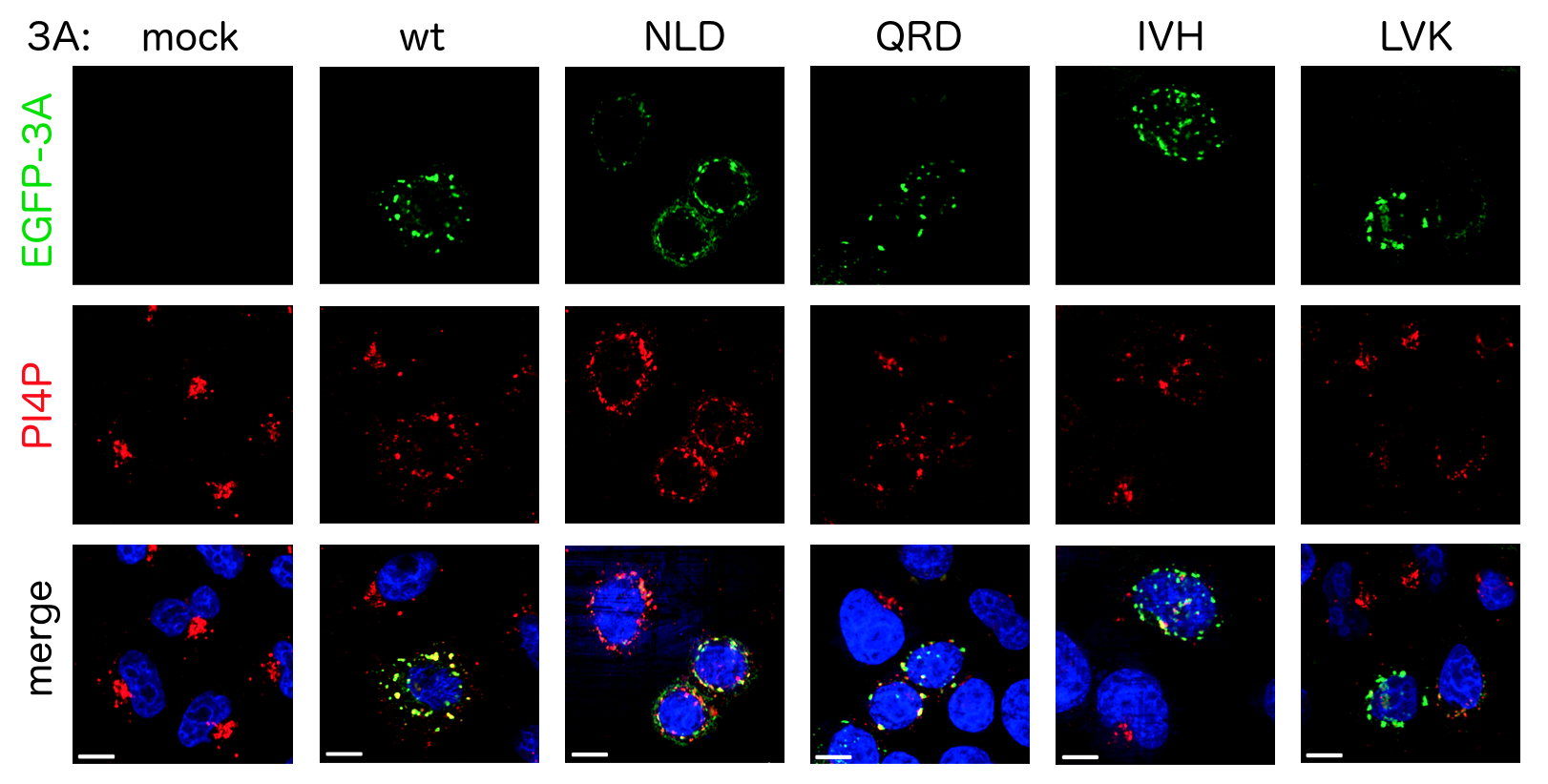

Supplement: S2 Fig — EGFP-fused wild-type EVD68 3A and its mutants were overexpressed in HeLa cells. The cells were fixed and immunostained with the anti-PI4P antibody (Echelon #Z-P004). Scale bars represent 10 μm. (TIF) [file ppat.1007962.s002.tif]

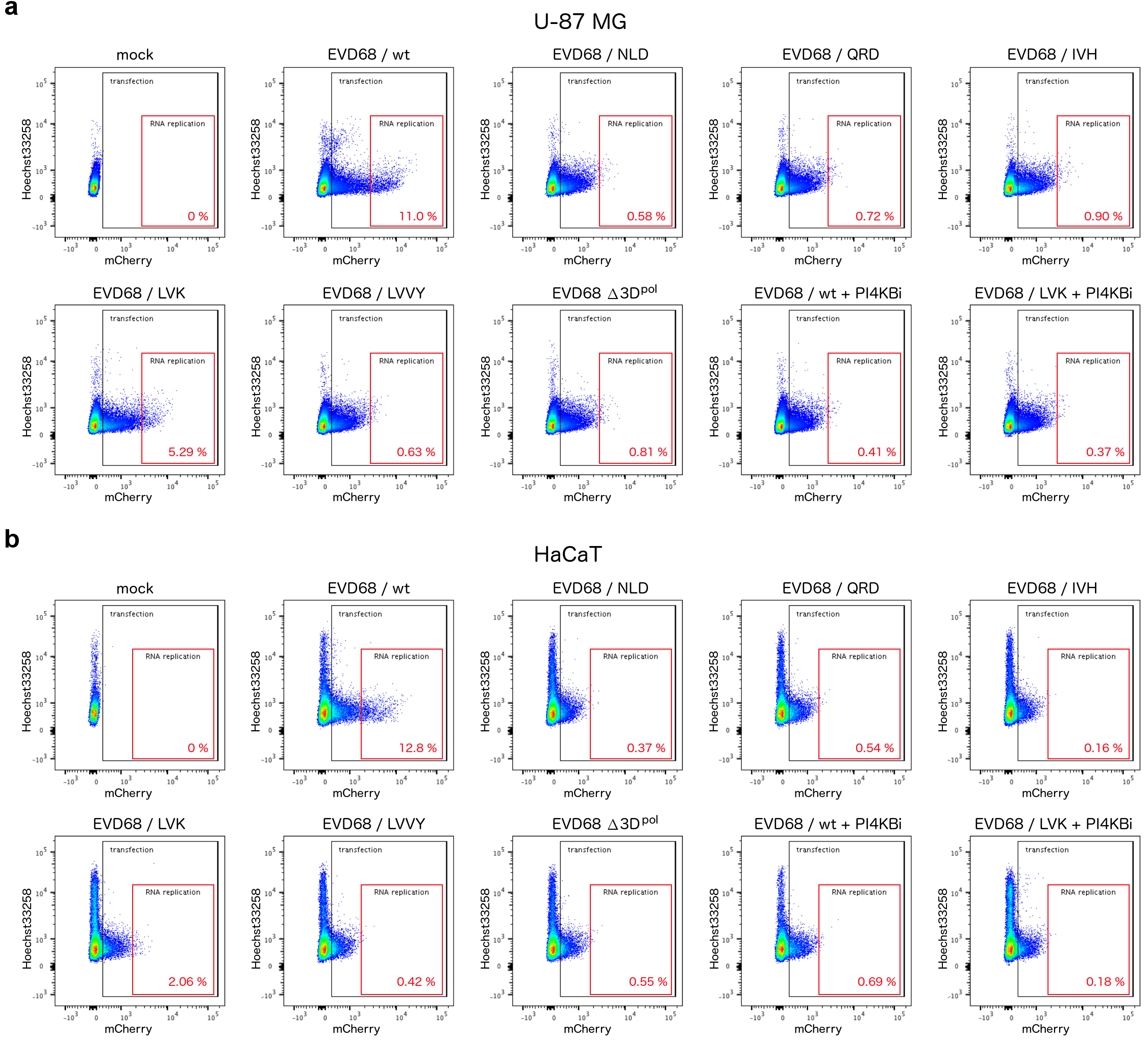

Supplement: S3 Fig — a-b, Human glioblastoma cells U-87 MG (a) or keratinocytes HaCaT (b) were transfected with the T7-amplified EVD68 Fermon strain subgenomic replicon wild-type RNA or its mutants as indicated, and the reporter mCherry fluorescence was determined by flow cytometry. Staining with the Hoechst33258 dye was added to determine the cell viability. The level of RNA replication was expressed as a percentage of cells with the mCherry signal above the threshold determined using the viral polymerase-lacking mutant Δ3Dpol (red region), further normalized to the transfection efficiency (black region). Data from one representative experiment are shown; please see Figs 3f, 3g and 5i for the quantification based on two independent experiments. PI4KBi, a PI4KB-specific inhibitor (compound 10 in Mejdrova et al. [10]). (TIF) [file ppat.1007962.s003.tif]

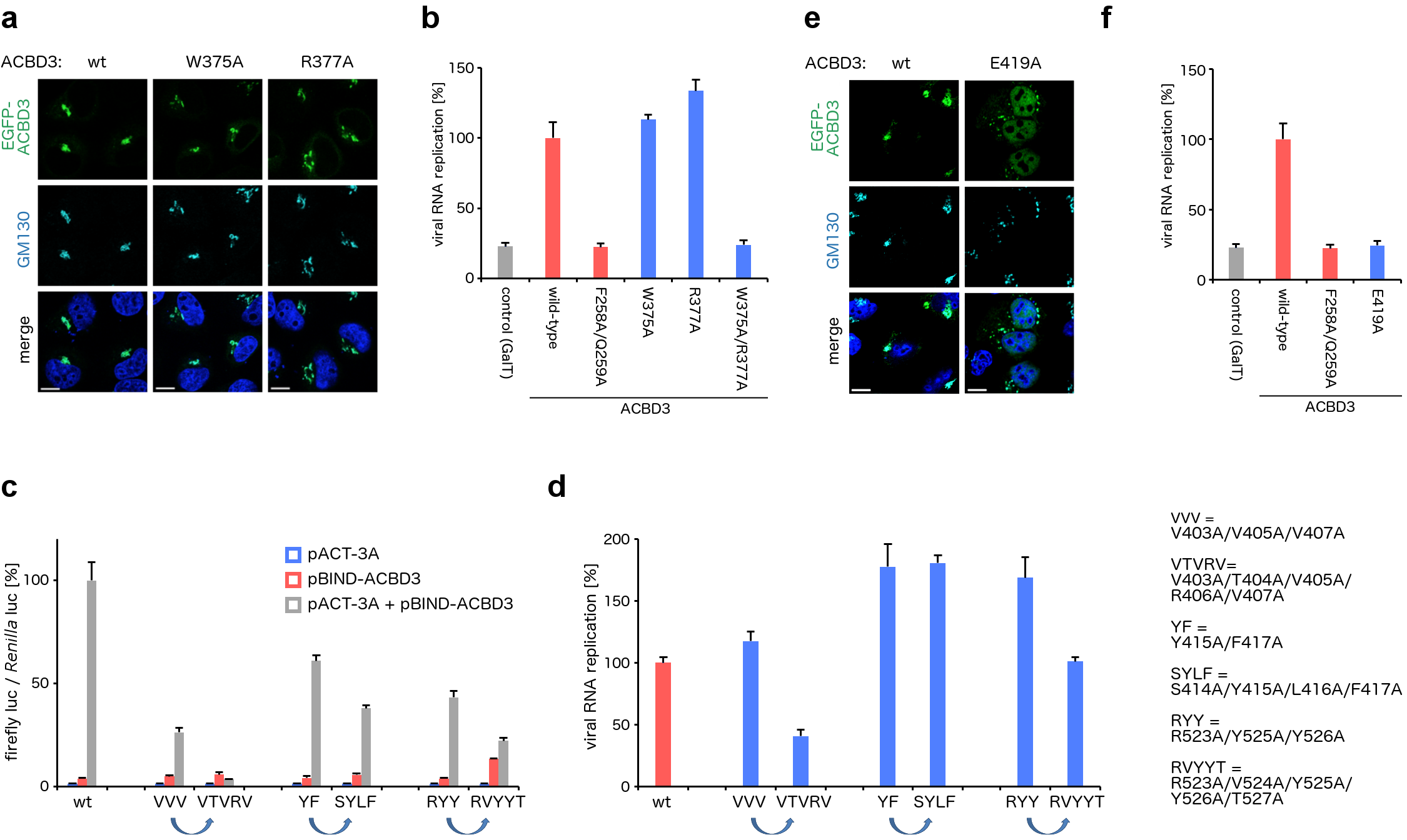

Supplement: S4 Fig — a, e, Localization of the ACBD3 mutants. EGFP-fused wild-type ACBD3 or its mutants were overexpressed in HeLa ACBD3 knock-out cells. Cells were fixed and immunostained with the anti-GM130 antibody (marker of Golgi). Scale bars represent 10 μm. b, d, f, Rescue of enterovirus replication by the ACBD3 mutants. HeLa ACBD3 knock-out cells were transfected with wild-type ACBD3 or its mutants, and enterovirus replication was determined using the Renilla luciferase-expressing CVB3 virus by the Renilla luciferase assay system. GalT and ACBD3 F258A/Q259A were used as controls. c, Mammalian-two-hybrid assay with the ACBD3 mutants and wild-type 3A. HeLa cells were transfected as indicated and the firefly luciferase activity normalized to the Renilla luciferase activity was determined using a dual-luciferase reporter assay system. (TIF) [file ppat.1007962.s004.tif]

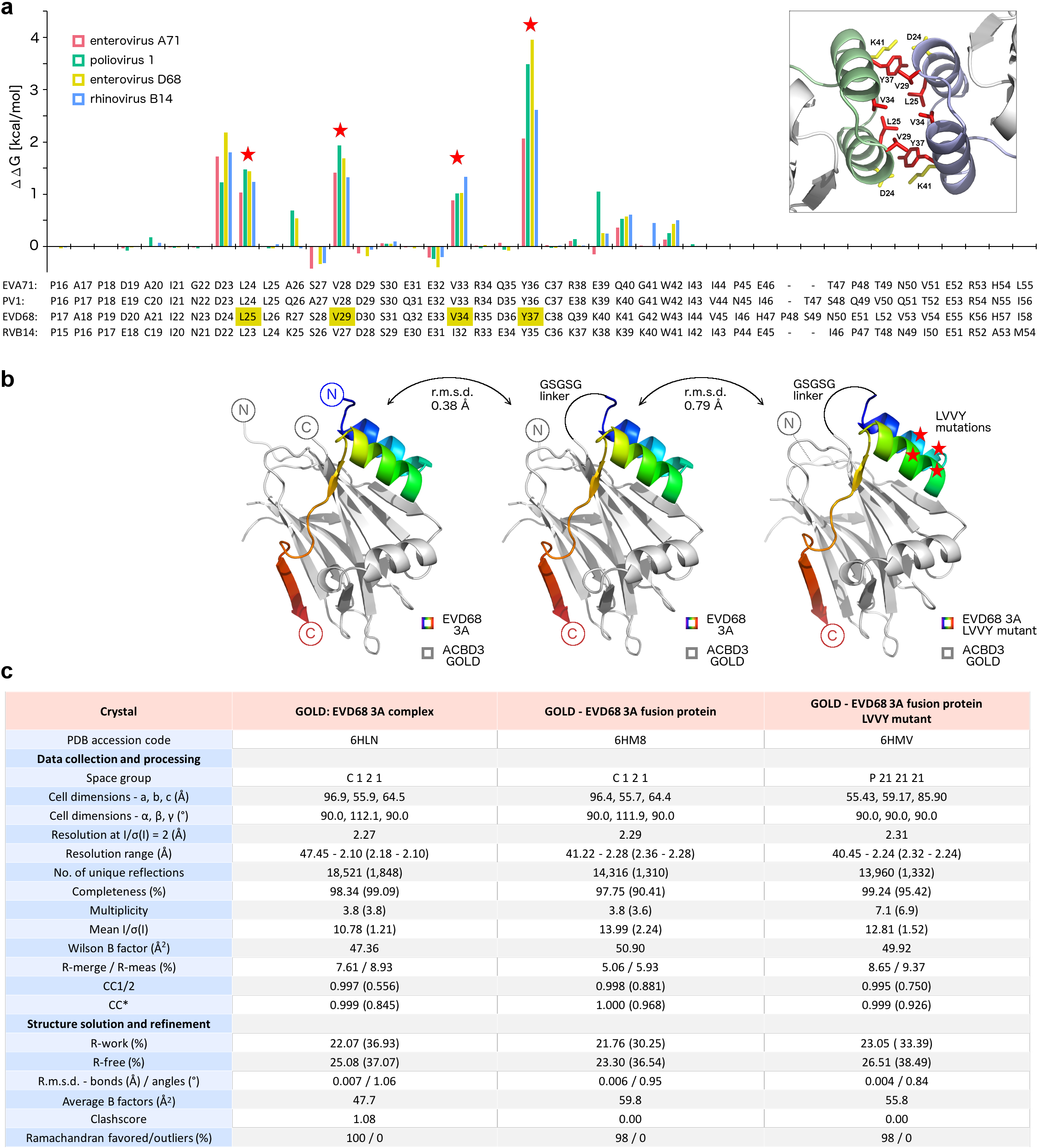

Supplement: S5 Fig — a, Changes of the dimerization energies of to-alanine mutants of the GOLD: 3A complexes as obtained with the Pssm tool of the FoldX software package [24] using the crystal structures presented in this work. Residues forming the hydrophobic core of the dimerization interface are marked by asterisks. In the inset, a detailed view of the EVD68 3A dimerization interface colored as in Fig 5a is shown. b, Overall fold of the GOLD: EVD68 3A complex formed by two individual proteins (left), wild-type GOLD—EVD68 3A fusion protein (middle), and its LVVY mutant (right). The ACBD3 GOLD domain is depicted in grey, the EVD68 3A protein in rainbow colors from blue (N terminus) to red (C terminus). Residues forming the hydrophobic core of the dimerization interface (mutated in the LVVY mutant) are marked by asterisks. c, Statistics for data collection and processing, structure solution and refinement of the proteins and protein complexes shown in (b). Numbers in parentheses refer to the highest resolution shell of the respective dataset. (TIF) [file ppat.1007962.s005.tif]

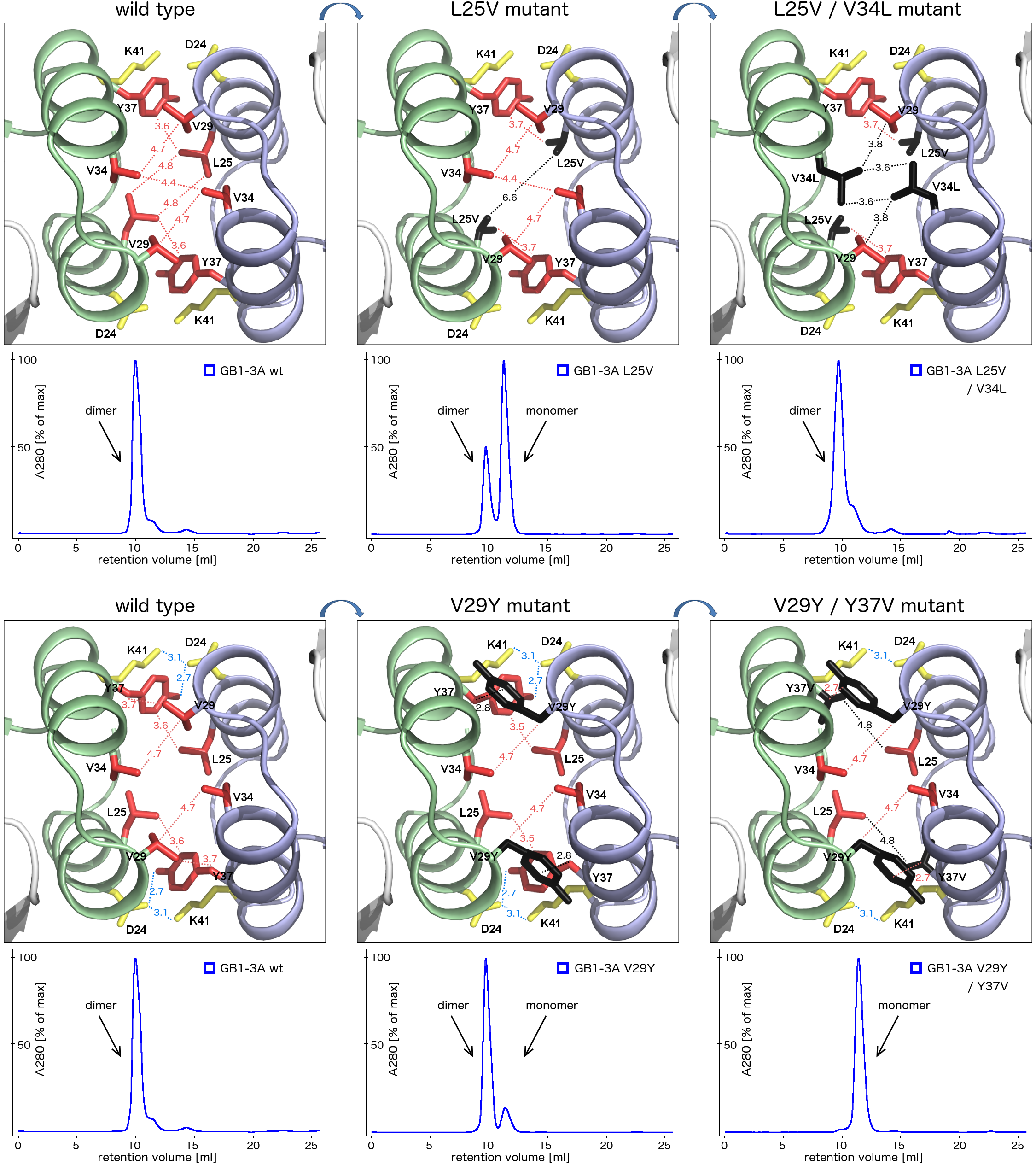

Supplement: S6 Fig — In the upper panels, a detailed view of the EVD68 3A dimerization interface colored as in Fig 5a is shown, except for the mutated residues, which are depicted in black. Distances of the closest atom pairs of selected residues are shown in Angstroms. Homology models of the mutant 3A proteins were generated by mutating the respective residues in Coot followed by the energy minimization in Swiss-PDBViewer. In the lower panels, elution profiles of the GB1-fused wild-type and mutant EVD68 3A proteins are shown. Each protein at a final concentration of 100 μM was analyzed by size exclusion chromatography using the Superdex 10/300 Increase column (GE Healthcare) and its elution was monitored by the absorbance at 280 nm. (TIF) [file ppat.1007962.s006.tif]

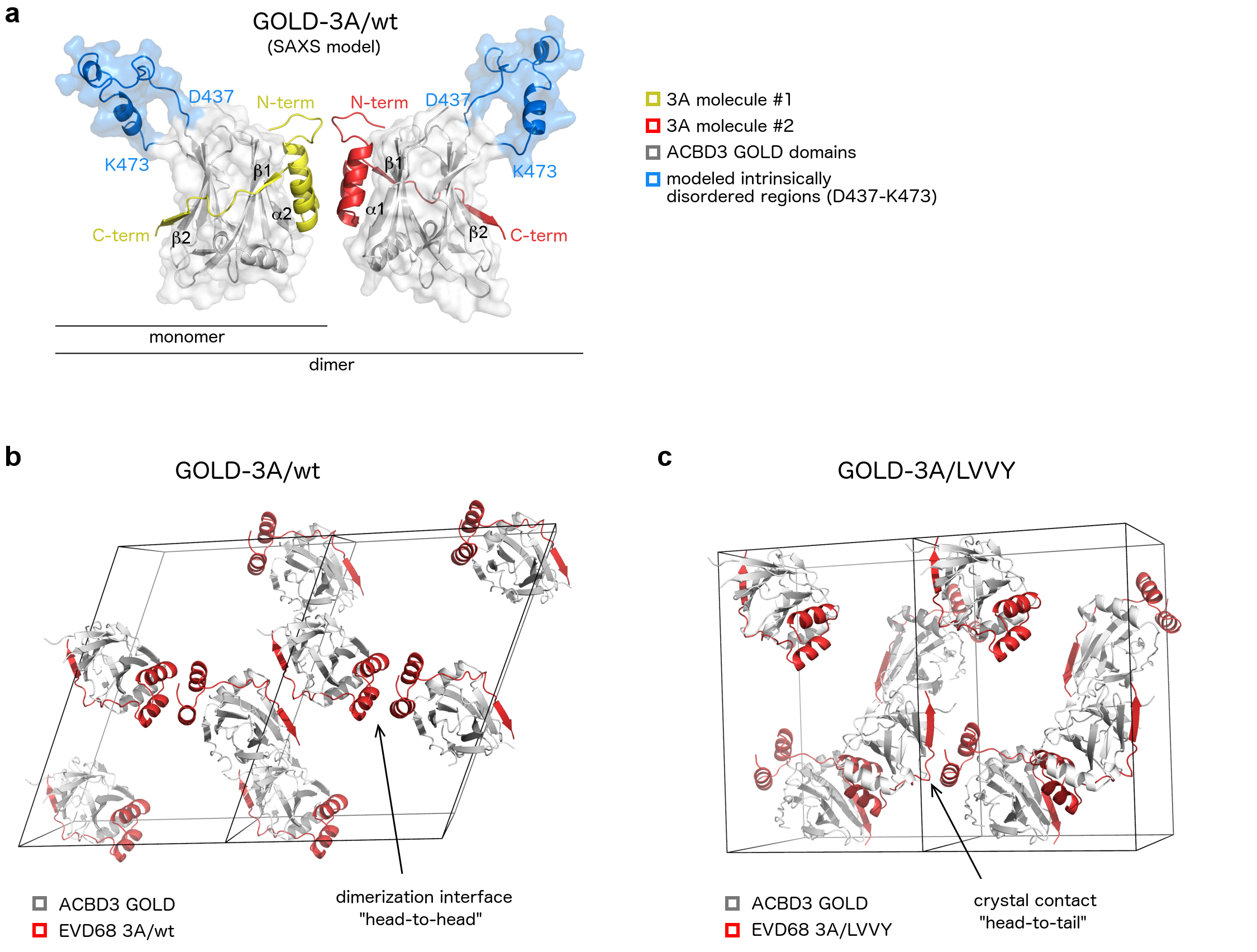

Supplement: S7 Fig — a, Structural model of the dimer of the GOLD—EVD68 3A fusion proteins used for the SAXS analysis. The ACBD3 GOLD domains are shown in cartoon representation with a semi-transparent surface and colored in grey except for the modeled intrinsically disordered loops of ACBD3 (D437-K473), which are depicted in blue. The EVD68 3A proteins are colored in yellow and red. b-c, Crystal packing of the wild-type GOLD—EVD68 3A fusion protein (b) and its LVVY mutant (c). The content of two unit cells with protein backbones in cartoon representation is shown. The ACBD3 GOLD domain is depicted in grey, the EVD68 3A protein in red. Wild-type 3A forms a crystal-packing contact through the 3A dimerization interface (b), while in the case of the LVVY mutant this contact is not preserved and instead, the N-terminal alpha helix of 3A forms a crystal-packing contact with the C-terminal beta strand of another 3A molecule (c). (TIF) [file ppat.1007962.s007.tif]

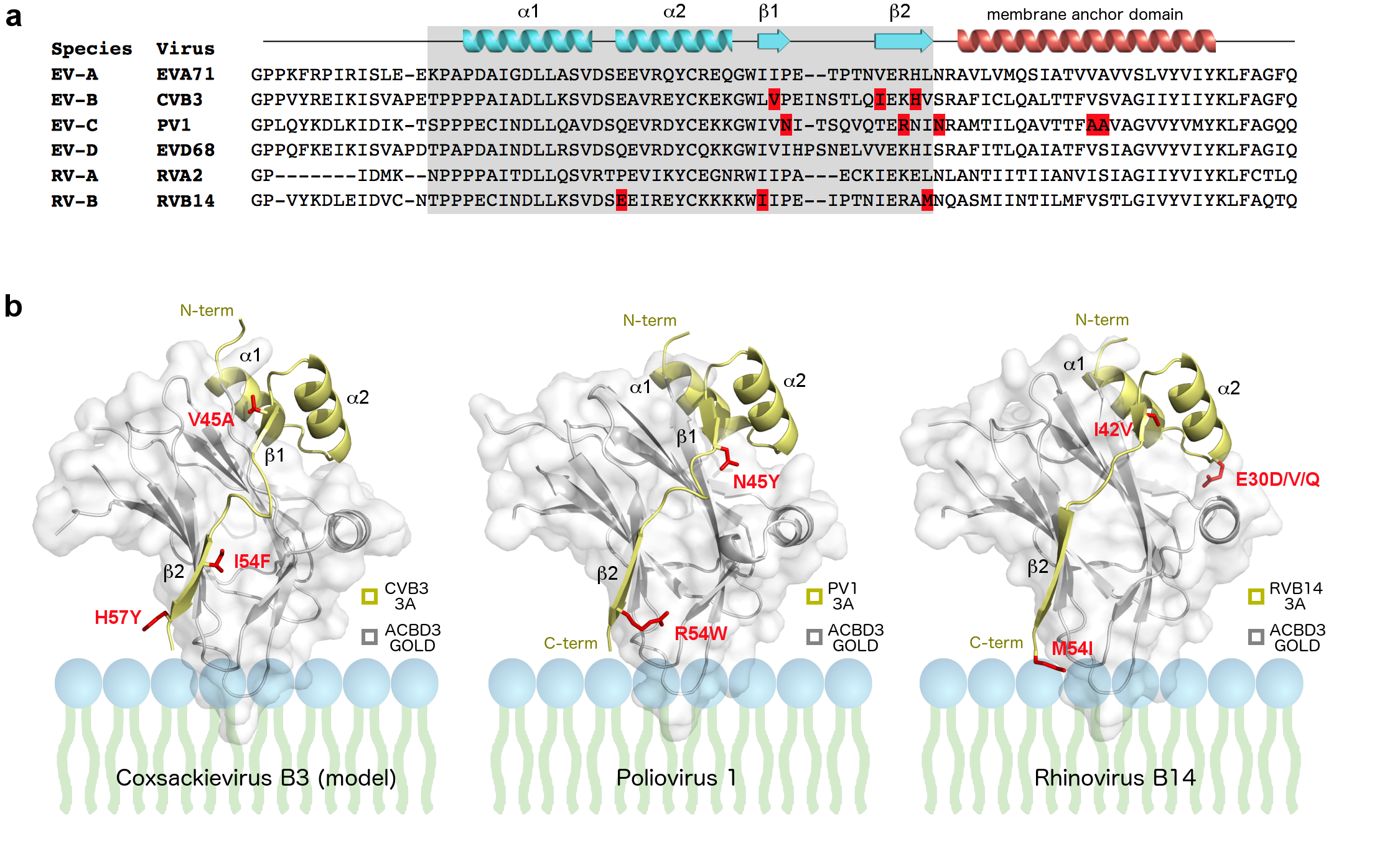

Supplement: S8 Fig — a, Localization of the PI4KB/OSBP-inhibition resistance-conferring mutations within the primary sequences of the enterovirus 3A proteins. Sequences of the 3A proteins of selected enteroviruses used in this study were aligned as in Fig 1a. Secondary structures present in the crystal structures of the ACBD3: 3A complexes (colored in light blue) and the hydrophobic alpha helix anchoring the 3A proteins to the membrane (colored in red) are indicated above the sequences. ACBD3-binding regions are shaded in grey. Residues whose mutations have been reported to confer resistance to the PI4KB/OSBP-specific inhibitors, i.e. PV1 N45Y, R54W, N57D, A70T, and A71S, CVB3 V45A, I54F, and H57Y, and RVB14 E30D/V/Q, I42V, and M54I [31–34], are highlighted in red. b, Localization of the PI4KB/OSBP-inhibition resistance-conferring mutations within the structures of the GOLD: 3A complexes. A homology model of the GOLD: CVB3 3A complex was generated by the I-TASSER server [58] using the crystal structure of the GOLD: EVD68 3A complex as a template. The ACBD3 GOLD domain is shown in cartoon representation with a semi-transparent surface and colored in grey; the enterovirus 3A proteins are depicted in yellow. Residues whose mutations have been reported to confer resistance to the PI4KB/OSBP-specific inhibitors are highlighted in red. (TIF) [file ppat.1007962.s008.tif]

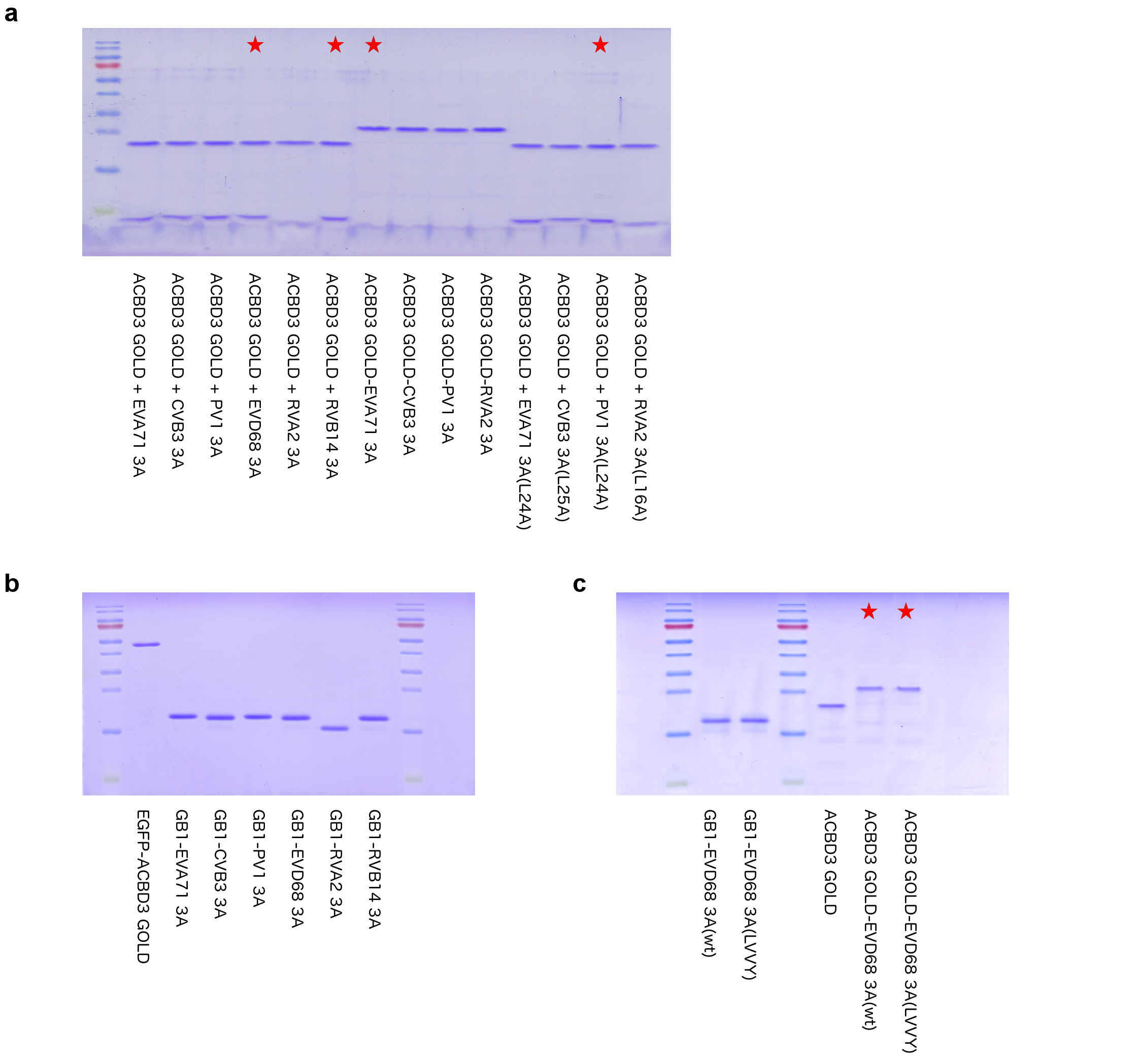

Supplement: S9 Fig — Recombinant proteins used for the crystallographic analysis (a), microscale thermophoresis (b), and SAXS analysis (c) were resolved by SDS-PAGE using the 15% polyacrylamide gels and stained with Coomassie Blue. The hyphen signs ("-") indicate fusion proteins, while the plus signs ("+") indicate complexes of two individual proteins. The uncomplexed viral proteins used for microscale thermophoresis and SAXS analysis were fused to the B1 domain of streptococcal protein G ("GB1 tag") to improve their solubility and to avoid their non-specific aggregation. Asterisks indicate the successfully crystallized proteins and protein complexes. (TIF) [file ppat.1007962.s009.tif]
